# Supplementary material for: Priority healthcare needs amongst people experiencing homelessness in Dublin, Ireland: A qualitative evaluation of community expert experiences and opinions
Source: PLoS One. 2023 Dec 14;18(12):e0290599. doi: 10.1371/journal.pone.0290599 (PMC10720995; doi:10.1371/journal.pone.0290599)
Supplement: S1 Table — (DOCX) [file pone.0290599.s001.docx]

**Table 3.** Priority Healthcare Needs Themes & Sub-Themes

| Level | **Theme**/Sub-Theme | Sample Quote |
| --- | --- | --- |
| Societal | **Promote Culture that Values Health Equity** | *“The priority is not for our guys. Look at the housing crisis, or the homelessness crisis. With time, it’s not getting better, it’s getting worse. The policymakers, the parties in charge, wider society… we tend to prioritise the most wealthy. We have to start prioritising those most in need.” (Addiction Services 2)* |
| Policy | **Accelerate Action in Addressing Health Inequalities** | *“In terms of health, long standing and sometimes chronic health issues may not be completely related to substance use. It’s related to poverty, housing, potentially domestic violence, tragic accidents, incidents… a conglomeration of issues that require additional support and back up.” (Researcher2)* |
|  | Strategic Planning | *“15 years I'm doing this now and there's been billions pumped into homeless and addiction services. I think there's about 55,000 people now working in the industry and yet, it's getting worse. It’s always reactive policies. It’s consistency we need and actually talking to people and thinking through long-term strategies.” (Addiction Services1)* |
|  | Housing First | *“You have to understand, these people have no homes. Their healthcare isn’t going to be a priority. There has to be more housing available for people in addiction because, I mean, how can you expect somebody to get off drugs who’s living on the streets?” (Addiction Services4)* |
|  | Social Support Options | *“A lot of health interventions, particularly for homeless people in hospital, are medication but a lot of the issues are symptoms. They're symptomatic of loneliness, loss, trauma, or whatever it is that life has thrown at them. Or the 'lack'. For a lot of people on the streets, there would have been a lack in terms of mothering, parenting...” (Psychotherapist1)*  *“The health service, if you're talking about physical health, hospitals, and stuff like that, it's quite medicalised and they deal with people as individuals. I'm very much a social worker that [believes in] community social work. I'd like to see more about that collaboration pieces with ourselves and community development workers, community development programmes, that kind of brings us very much back into the communities. As things stand, we tend to parachute in.” (Social Care1)* |
|  | Interagency Collaboration | *“If you think about it, clients don’t know what’s happening with support services when they’re dealing with them all in different silos. When they all come together and actually have a meeting, they’re much more informed. All the other services are also much more informed. They’re seeing the person in a much broader perspective.” (Social Care1)* |
|  | Data sharing and Linkage | *“That would be the goal, that that would be the dream to have the electronic patient record that follows the patient and makes healthcare seamless. But no…Often people get the wheel recreated, they start an admission again from scratch. They’ll get the same investigations done; the same treatment initiated for the original duration…there’s a bit of repetition”.* (Hospital2) |
|  | Auditing | *“Case managers should be involved in every single aspect of someone’s care. All of it. Now we’re seeing people being called case managers but they’re actually key workers, maybe they just deal with addiction. Clients end up in court and the judge sees they’ve had case management for a year and it’s not working, but they haven’t. Nobody’s looked after their mental health…” (Addiction Services3)* |
| Health Services | **Remove Barriers to Access** | *“Primary care is set up for people with addresses. It's not set up for people in homelessness. Then the current hospital system for people in homelessness doesn’t work. It's never their priority, their healthcare. Their priority is where they're going to sleep tonight, where they're going to get their next meal, where they're going to get money for their next week of drugs. So, coming to a busy public hospital and sitting in a waiting room for hours on end… Then if a person in homelessness is for discharge, they’ll hand them a prescription for a long list of medications. They’re not going to check, ‘Have they a medical card? Have they money to get it?’ Unfortunately, due to the business of the hospital, they treat them like everyone else.” (Hospital3)* |
|  | More mental health, addiction, women-centred, and GP services | *“Women were saying, ‘Look, I have a personality disorder, but apparently that's not a Category 1 illness.’ So, women were really struggling with their mental health and trying to reach out, but the services were insufficient.” (Researcher3)*  *“People are out in the community fighting to get drug free and to go into treatment. The whole idea is that when they say they want to do something about this, let's get them into residential treatment straight away, not be on waiting lists for months.” (Addiction Services1)*  *“Ideally, we would work with homeless people and as soon as people have an address, we would try and get them into a regular local, practice in their area. For instance, I have a girl today who's pregnant and due a baby in two months’ time. She needs to have a GP do the baby's vaccines and the baby's development. We couldn't do any of that [here]. There's a practice that I'd like her to go to. She now has a home, but they're full.” (Homeless Health Services4)* |
|  | Safe Services | *“People who mightn't use [that low-threshold health service] are people who are trying to stay off drugs because there's a lot of drug use around and that's really difficult to navigate. A lot of women might feel intimidated, people who aren't drug users might find it intimidating, and then there's also often a problem for some of the people we work with that they owe drug debts. Sometimes they can't go to services because they are worried that somebody there will know them and be looking for them to beat them up or kill them.” (Hospital1)* |
|  | Resolved Care Pathways | *“Mental health services and drug services have always ping ponged off each other. ‘Oh, it's not a drug issue, it's a mental health issue. It's not a mental health issue, it's a drug issue.’ All these families that fall between the stools of other services come to us and it becomes a child protection issue, but it's not. It's a disability issue. It's a mental health issue. It's a substance use issue from the parents.” (Social Care1)* |
|  | Trauma-Informed Education and Training | *“In our lectures, there was nothing about ‘How does an addiction emerge? What is its relation to socioeconomic circumstance? To gender? To violence? To age?’ Look, it’s a societal thing, but for people who were absolutely going to come face to face with substance use, we were not taught about it, and it was only once I left college that I started to learn about terms like harm reduction, methadone, exploitation, trauma.” (Researcher3)* |
|  | Expanding Inclusion Health | *“I saw a guy today who has a big knife slash wound and it's caught his nerve and he's got nerve pain. We made several appointments and we even had people meet him to bring him to appointments and he still misses the appointments because he can’t be found. He's sleeping rough. But the inclusion team get that. They completely understand, and they will give someone a cup of tea when they arrive and give them an appointment soon after if they miss one. Whereas if you were dealing with the general hospital, a missed appointment means back down to the end of the list”. (Homeless Health Services4)* |
|  | Outreach Programmes and Peer Support | *“Wi*th Hep-C, w*e were able to break away from that really traditional, kind of patriarchal view of how medicine should work, and we got the treatment out to the patients in the community in a way that really worked for them.*.*. With epilepsy, chronic obstructive pulmonary disease, even HIV care, those are things that really take a lot more long-term management than the HEP-C programme did. We need to be able to think outside the box a little bit in how we're delivering that healthcare to patients…it'll be new ground. It's not an established thing.” (Hospital2)* |
| Research | **Address Knowledge Gaps** | *“I'm very aware that I only know about the people that come to us and the people who don't come to us don't trust us, probably, and I don't know anything about them because they don't come to us.” (Homeless Health Services2)* |
|  | Optimal Addiction Care and Support | “*I’m fascinated at the gap in knowledge or belief between doctors and those in addiction, and maybe we have different ideas of what would be the best path because we don’t actually have the same aim in mind. Are patients seeking stability… would they ultimately like to be drug-free, or something else? Are we having honest conversations with patients in which they feel they can share what their preference/their treatment goal would be?*” (Homeless Health Services1)  *“We have patients who have - because of their addiction - sleeping difficulties, - because of their hostel - sleeping difficulties. If you were to prescribe sleeping tablets, they would be on them forever and that is what I know because I'm 20 years doing this, and I cannot get people who are on sleeping tablets off of them. So, for all of us, there's a problem. I don’t know what the answer is, but I know the whole benzodiazepine scene has changed utterly from pre-COVID in that most of our patients are on five Benzodiazepine three times a day now, or a benzodiazepine detox. The ones who are on five three times a day are doing much, much better. That's what I can see. They're not buying handfuls or presenting drug affected.” (Homeless Health Services4)* |
|  | Healthcare Access Amongst Vulnerable Populations | *“[Asylum seekers] are landing in Ireland, they register at the International Protection Office, and they're told, ‘There are no beds’. This is very new now as in the last six weeks to two months. I'd a man who was diabetic and had to tell him to sleep on the street on that freezing cold night. Maybe a month ago, three weeks ago, on a Thursday, I remember. And he kept on asking on his Google Translate code, ‘But where am I to sleep?’ and I had nothing. We never did that before.” (Hospital3)*  *“For me at the moment, the thing that worries me the most are people entering homelessness with intellectual disability or the ones leaving foster care. If you picked one of one of those groups and looked at their trajectories and how the services are set up to access them. I think that if society as a whole was really aware of what's going on, I think that there would be, there could be change in that system.” (Hospital1)* |
| Individual | **Include homeless service users in health and social service design, planning, and delivery** | *“I'm thinking of my HIV outpatients or Hep C outpatients. If somebody makes them do it, or prompts them to come, they kind of just do it to keep you happy rather than doing it to look after their health. They can't usually choose what food they eat. They can't choose their living environment. There's very little control over their health anyway...There’s a sense of not being entitled to anything better.” (Hospital1)*  *“We really don't have any data that looks at how people who are in homelessness, or in socially marginalised groups would like their healthcare, or think is the best way for their healthcare to be delivered. So that obviously we're not just making all the decisions for them.” (Hospital2)* |
