# Supplementary material for: Priority healthcare needs amongst people experiencing homelessness in Dublin, Ireland: A qualitative evaluation of community expert experiences and opinions
Source: PLoS One. 2023 Dec 14;18(12):e0290599. doi: 10.1371/journal.pone.0290599 (PMC10720995; doi:10.1371/journal.pone.0290599)
Supplement: S1 Text — Anonymised excerpts from semi-structured interview transcripts. (PDF) [file pone.0290599.s002.pdf]

ABOUT THIS DOCUMENT

The document contains transcript excerpts justifying each point made in the PLOS ONE manuscript entitled, *"Priority healthcare needs amongst people experiencing homelessness in Dublin, Ireland: a qualitative evaluation of community expert experiences and opinions."*

Transcript excerpts have been fully anonymised. Results are in black and transcript excerpts in colour. Colours are assigned based on occupation: Homeless Health Services, Hospital, Addiction Services, Government, Social Care, Psychotherapy. For any questions, please contact carolyn.ingram@ucd.ie.

You may not affix your names to the enclosed data.

**Date of Interviews**	September 2022 – April 2023
**Semi-Structured Interview Questions**	•What does your work currently focus on and with what populations?<br>•What changes have you seen take place in the community and the services you provide?<br>•What programmes do you see working well? Or less well?<br>•What changes would you make if you could?<br>•Have you spotted any gaps in services?<br>•Are most homeless people using provided services?<br>•Which health issues are of most concern to the communities you work with?<br>•What are the strengths and assets in these communities?<br>•Where do we need more evidence?<br>•What would you focus your next research project on?
**Anonymisation**	Where we have changed or removed a word or phrase, we have either put brackets and italicized the new text [*New name*] or marked [*Retracted for confidentiality*].
**File format**	PDF
**Description**	A purposive, criterion-i/snowball sampling strategy was used to identify professionals working in homeless health and/or

addiction services in Dublin, stratified by occupation type. Potential community experts (CEs) were identified through an internet search of homeless health and addiction services in Dublin. Interviewed CEs were invited to recommend colleagues they felt would have relevant perspectives on community health needs. Semi-structured interviews were conducted between September 2022 and March 2023 utilising ZOOM™, the phone, or in person according to participant preference. The interviewer has formal qualitative research training. CEs were presented with an information sheet and gave audio recorded, informed oral consent – considered appropriate for remote research conducted with non-vulnerable participants 18 - in the full knowledge that interviews would be audio recorded, transcribed, and anonymised as approved by the researchers' institutional Human Research Ethics Committee (LS-E-125-Ingram-Perrotta-Exemption). Interview questions were based on World Health Organization Community Health Needs Assessment guidelines and refined based on the lead researcher's field work with homeless service users.

Transcript excerpts included from the following interviews:

**Pseudonym**	**Professional Role**	**Location**	**Interview Format**
Homeless Health Services1	Healthcare professional (Primary care)	Drop-in primary and addiction care	Zoom
Homeless Health Services2	Healthcare professional (Primary care)	Drop-in primary and addiction care	Zoom
Homeless Health Services3	Healthcare professional (Primary care)	Drop-in primary and addiction care	Zoom

Homeless Health Services4	Healthcare professional (Primary care)	Drop-in primary and addiction care	In person
Homeless Health Services5	Healthcare professional (Primary care)	Drop-in primary and addiction care	In person
Hospital1	Healthcare professional (Hospital)	Hospital Inclusion Health service	Zoom
Hospital2	Healthcare professional (Hospital)	Hospital Inclusion Health service	Zoom
Hospital3	Healthcare professional (Hospital)	Hospital Inclusion Health service	Zoom
Government1	Research and Data Officer	Health Service Executive Ireland	Zoom
Government2	Project Manager	Health Service Executive Ireland	Zoom
Psychotherapist1	Psychotherapist	Drop-in primary and addiction care	In person
Researcher1	Researcher and Physiotherapist	Drop-in primary and addiction care	Zoom

	Researcher2	Researcher	Residential treatment facility	Zoom
	Researcher3	Researcher	Drop-in primary and addiction care<br><br>Emergency department	Zoom
	Social Care1	Child and Family Support Network Coordinator	Government Child and Family Agency	Zoom
	Addiction Services1	Programme Coordinator	Drugs and Alcohol Task Force	Phone
	Addiction Services2	Team Leader	Dublin Harm Reduction NGO	In person
	Addiction Services3	Management	Residential Treatment Facility	In Person
	Addiction Services4	Healthcare professional	Residential Treatment Facility	Phone

State of homelessness & addiction in Dublin

CEs described the changing landscape of homelessness in the city. Though a ban on evictions reduced the number of people entering homelessness during the Covid-19 pandemic, housing statistics show a sharp increase as soon as the ban was lifted (Government1).

> "You could see how, for example, during COVID-19 pandemic, there was a fall in the number of people entering homelessness because there was a ban on eviction. But then soon after, you can see a massive increase. Also what became apparent from the things we were hearing on the ground and as well from data analysis was that the current landscape of homelessness in Ireland is changing." (Government 1)

The severity of the housing crisis has resulted in emerging homelessness unrelated to addiction. More migrants and asylum seekers, youth leaving social care, elderly individuals, women, and families are entering homelessness (Government1, Hospital1).

> "So, we do have more migrants entering homelessness than in the past, we have more women entering homelessness. We have young people entering homelessness, but at the same time the number of older people who are entering homelessness is increasing as well. So all that if you want to call, I heard this term 'global homeless' in a way about people who are refugees and then we have clients in direct provision as well and sometimes it might happen that they might end up in homelessness as well". (Government 1)

> "We're seeing some - with the ongoing housing crisis - we're seeing some elderly people sometimes with dementia going into homelessness because they can't find accommodation. Where they've lost their rental property because they can't remember to pay their rent because they have dementia. And that's a really bad idea. And the disabilities services are another real pinch point where there are a lot of people with intellectual disability who are in homelessness because of their intellectual disability and they really do badly, and they shouldn't be there. It's not fair." (Hospital 1)

For the first time in 2023, newly arrived asylum seekers are being discharged from hospital with no place to sleep due to a shortage of beds in homeless accommodation (Hospital3).

> "Yeah, just because before they were always getting accommodation and had their own support. But now they're landing in Ireland. They register at the International Protection Office and they're told, "There are no beds". I think they're given an 18 Euro voucher for Dunnes and told to sleep on the streets. This is very new now as in the last six weeks to two months. So, I've had to send people,...I'd a man who was diabetic and had to tell him to sleep on the street on that freezing cold night. Maybe a month ago, three weeks ago, on a Thursday, I remember. And he kept on asking on his Google Translate code, "But where am I to sleep?" and I had nothing. We never did that before. So, the situation is getting worse and worse." (Hospital 3)

CEs noted that the nature of drug addiction is also changing. Today, most substance misuse in Ireland involves some combination of alcohol, cocaine, benzodiazepines, cannabis, and opiates. CEs primarily treat and respond to this type of polydrug use: *"a lot of people will probe back into heroin and [benzodiazepines] to come down from a crack cocaine binge. Very rarely I've met somebody who was going to the clinic and only taking methadone."* (Addiction Services1)

> “A lot of people will probe back into heroine and tablets to come down from a crack cocaine binge. Very rarely I've met somebody who was just going to the clinic and *only* taking methadone. So, they've now spiraled into polydrug use which is a huge thing in Ireland. It's not really recognized, people try to put labels: "Well there's an opiate user, there's a benzos user." It's polydrug use. I've -- they're few and far between. Maybe ten people. But yet, the policies are in place for that whereas the case management piece in the clinics is actually highlighting the fact that, "no actually, these are polydrug users." Then again it was laziness on behalf of a lot of people,...a lot of people doing the assessments would just write heroine. You know, you get an option for six or seven different drugs and then there's an option for which is the problem drug. So somebody can be taking heroine or taking methadone and it's not a problem. But, the problem drug is benzos because they're walking around like they're zombies.” (Addiction Services1)

> “5% of the substance user in Ireland at the moment would be opiates. The majority would be alcohol, cocaine and benzos. And cannabis. Yet, there's no real policies for that. They're only starting to come around to that now.” (Addiction Services1)

Crack cocaine use has skyrocketed and is a particularly difficult problem as there are few effective treatment options (Homeless Health Services4, Addiction Services1).

> “Well, the evidence is there. Among the homeless, because it's cheap and cheerful - I'll use the description from one of my clients - you know it's 10 quid, it does exactly what it says on the can. People on the street are looking for the quick, 'Get out of this horrible misery'. And crack cocaine is the one that does that.” (Addiction Services1)

> “Let's say someone is a polydrug user. He's taking heroin. He's taking tablets and benzodiazepine and crack. Crack is a big, big problem in Dublin.” (Addiction Services2)

> “The nature of drugs being misused has changed and crack cocaine has become a really big problem. It's really, really difficult to manage at the moment. There isn't, obviously, things we can do to help get people off crack cocaine, and the really most effective treatment option is to get somebody in for stabilisiation. Into a stabilisation residential admission.” (Homeless Health Services 4)

> “Crack cocaine explosion. Very little treatment for that.” (Addiction Services1)

> “The nature of addiction is changing. Crack cocaine is more prevalent now.” (Addiction Services3)

For other substances, Dublin’s addiction care services offer a mixture of opiate substitution – mostly methadone with some suboxone prescribing –, alcohol detoxification, benzodiazepine detoxification, and more recently, benzodiazepine maintenance.

> “So, what we would see here is a mixture of opiate substitution, some methadone, mostly methadone, some suboxone, some alcohol detoxes, some benzodiazepine detoxes,” (Homeless Health Services4)

> “Broadly speaking, we have 3 categories of people: [1] People who don't get any benzodiazepines, which is most people, [2] people who are doing a benzo detox, so they're

on a particular schedule of reducing doses every two weeks, and then [3] some people who are with us for methadone, so they have to be coming every two weeks anyway to see us for methadone, some of those people will be on a maintenance dose of diazepam. And usually they're long term methadone patients who have tried to detox maybe more than once and just get to a certain stage, maybe around 10 or 15 milligrammes a day, and just below that they fall to pieces." (Homeless Health Services1)

Healthcare usage & outcomes

Patterns of healthcare usage by PEH have been widely documented. CEs reiterated that when in homelessness, immediate health needs (e.g., addiction, housing, acute injury, infection) take precedence over long-term preventative needs.

"In terms of preventative medicine, I mean it is interesting. I think what happens to homelessness because you're so focusing on the immediate health needs - so, for example, drug addiction obviously is an immediate health need, then you go on to getting housing, and then they often have poor health, like they've infections that they're managing, et cetera - that sometimes you can less focus on the important,...the long-term preventative needs. So, for example, I think that you'll find that they're almost all smokers. Very high rates of smoking. They have, you know,...cervical smear uptake, breast screening uptake would be poor. So, definitely there's gaps in in prevention." (Homeless Health Services3)

PEH have lower rates of medication adherence, tend to see the GP mostly for methadone, and to otherwise present to health services as a last resort.

"More of what I'm hearing is that people attend medical services when it's an emergency, you know, unscheduled care rather than waiting for appointments... The concept of looking after your health to live longer, and to live a healthier life, I just don't see it as something of concern. Just like, you know, people that engage in the exercise classes seem to really, really enjoy it and get a lot out of it. But even for that to be remembered, to go and do it again and see the benefits of it, has been really challenging. Someone else said, 'To be honest, I would choose taking cocaine sitting in my car and taking drugs over going to an exercise class. You know there's no competition.' (Researcher1)

"I would say a lot of people, well, a lot of people that I have spoken to would attend their doctor purely for methadone, to get their prescription. But they would be very clear that that's all that they're *allowed* to attend for or would have access to. I would say people generally say they never go to the doctor, they never get to see the doctor, and if they did, they just have negative things to say about healthcare or some would say they never need to because they're not,...You know the reasons why I would go to the doctor, sometimes they wouldn't see that as a need. I don't think their health would be a priority unless it was limiting their immediate health or getting from A to B, or if they had acute pain. I don't think their health would be valued in the same way. I would say people don't access services as much as they should or could. I'm just thinking of some people that would say that they had hypertension but may not renew the prescription. Even one particular participant forgot to take their medication that day. That would be a concern to me, but not to them, you know. So, I suppose I see that in that they don't view their ill health or their health conditions as something that needs to be

addressed right now. They don't see it as,...You know, primary prevention is a warning that this could get worse." (Researcher1)

"Let's say you have a rash and you wake up and you're withdrawing. Then yeah, priority is to get your heroin or buy some methadone on the street or something like that. Then by the time you raise some money, however you're gonna do that to get your heroin, and then you do that. Then you feel better so your rash is less of a priority for you anyway and then by the time you get around to kind of feeling like, *"oh, I should look into that again,"* maybe the morning clinic is closed. You know we're open as a walk in, so you try to remove the barriers but there are still barriers there. Inevitably, you know, you'd love to have a service that's walk-in all day but with staffing being what it is...But yeah, people just kind of think if the doors aren't open when it occurs to them and it's not easy enough for them to do it at that time, the barrier maybe looms larger for them than it would for someone else, you know." (Homeless Health Services1)

"The public perception is, "Oh they're just using the hospital for nothing." And I'm like, "Uh, no". Like I had one guy who broke both his hips and never came into hospital, like walked around on a broken hip at home. Can you imagine the pain? We only found out about it years later when he was in for something else. And he's a Traveller who's homeless since he's 11, who has alcohol dependence. So, you can really see those layers building up there." (Hospital1)

Internal barriers to presentation include mental health conditions, distrust of the health system, and internalised stigma, shame, and embarrassment.

"A lot of distrust of the health system, and actually more than distrust of the health system, I think there's a lot of internalised stigma and shame and then a lot of shaming and stigmatisation goes on in the healthcare setting. So that makes people very reluctant to use it." (Hospital1)

"It's important to know about why people didn't come as much as why they did come. Some reports have been around anxiety and embarrassment, which is a shame. I think that is also about not trusting people, perhaps. Anxiety and depression are commonly reported as well in this population. They're all so interconnected. I think that kind of mental health, and the anxiety and the depression that I am seeing, the high rates of it in this population has made it very hard for them to engage with healthcare." (Researcher1)

External barriers, particularly at the hospital level, include not getting methadone or Librium in a timely way, lack of freedom – particularly for those who have been in prison –, and judgement and blame from healthcare providers.

"There are multiple things happening here. There is stigma, there's anticipated stigma, there is impatience, there is a sort of situation of 'nobody's fault' in some ways. I guess I would say a couple of things. One, maybe most importantly: No, there is stigma happening and there absolutely is stigma happening around drug use. It's not uncommon to meet a doctor that doesn't [know about drugs], and so they don't view that as a category of illness that's worth treating." (Researcher3)

"So for instance, one of my informants overdosed, was taken to the hospital, overheard when she came round a couple of days later, one of the nurses saying sort of derogatory things about her methadone. Saying, "Oh, look, she's just gonna have to wait. She's just gonna have to..." I can get the the direct quote out of my notes, but like, "You know, look, we're not rushing on that, she might have to wait". And she was so mortified that they were talking about her in that way that she left. She left with the catheter bag still on her. Now, that's put down as a DAMA [Discharge against medical advice], that's put down as a, "Oh, she couldn't hang around. She was so desperate for the drugs she couldn't wait." Without sort of understanding. Actually, there's so much research now coming out saying that actually mismanagement of withdrawal is a key reason that people can't and don't stay in hospital because that experience is so difficult. It's not just that,...Oh my goodness. This lady was basically in pain and it wasn't being addressed in hospital. And a sort of way we think about methadone is, we're so scared of it that we're not like, "Well, what does it do to actually have a functional regime around that?". The other thing that my informant talked about in that situation was that like, she was *so* mortified that they were talking about her in those terms. And then she had to sit there and receive care from where she was feeling judged." (Researcher2)

"There's a big fear that they won't get their methadone or Librium in a timely way. And that's true. So, you don't get those until you're admitted to hospital. So, when you're in the emergency department being assessed and waiting for that process to happen, you don't get those things, and that can be like 24 hours. And maybe if you were sick, you weren't able to go to your methadone clinic the day before. So people can really feel really sick, really withdrawn. So, that's one reason. I think they're afraid of being shamed. And patients do get told, "Look what you've done to yourself. You're disgusting." Like *in those words,* and then also nonverbally. So that can be a barrier. Then, often, they don't like, they may be afraid - and it's true again - that they won't have the same freedom. So they won't be allowed to go out for a cigarette. They won't be allowed to have a drink, or whatever. All of those things mix together, I think, and a lot of them have had previous,...have been in prison. So, hospital can be really reminiscent of prison. That feeling being cooped up. So there's lots of barriers." (Hospital1)

"So, where I work, there's a lot of judgement that *[women facing addiction]* feel. So, they have come in with this perception to us that they're being judged already." (Addiction Services3)

Participant Hospital2 noted a tendency for transience across services. PEH may struggle to sustain a hospital admission due to their addiction or having co- diagnosis of a psychiatric disorder.

"It's a very transient kind of population in that we might start their treatment for infection. Some people can't sustain an admission for multiple reasons,...maybe due to them being very active in their addiction or having co- diagnosis of some psychiatric disorder where they just can't sustain an inpatient admission for the duration necessary. So we might start their treatment as an inpatient and for whatever reason the admission doesn't work out and they may go to James's hospital on the other side of the city or Beaumont or somewhere and have another part of their treatment there. So, they do move around healthcare quite a lot, and it

would be the same kind of group of people, but they would move to different healthcare institutions like that." (Hospital2)

CEs working in drop-in primary care perform mostly acute medicine, often related to addiction.

"So, what we would see here is a mixture of opiate substitution, some methadone, mostly methadone, some suboxone, some alcohol detoxes, some benzodiazepine detoxes, some acute medicine to do with addiction, primarily management of ulcers or cellulitis or trauma. That's acute general practice. What we don't do here is sort of chronic disease management, like diabetes or stuff like that. But we'll do the acute side of things, yeah." (Homeless Health Services4)

They see high rates of blood borne infectious diseases (i.e., HIV and Hep C), nutritional issues, psychiatric anxieties from taking street tablets, and physical – often injection-related – injury such as ulcers, cellulitis, or trauma.

"From an infectious disease, inclusion health crossover, what do we treat most? It would be skin and soft tissue infection from people who inject drugs. That would be a particularly high risk activity for skin and soft tissue infection. So, we would see a lot of that. And then blood borne viruses, so predominantly hepatitis C, but also HIV. Those would be the main things." (Hospital2)

"So yeah, I actually... I mean, you see a lot of addiction issues. Whether that's,...addiction's a big bracket, but kind of psychiatric related to addiction but also physical, you know, kind of injection related. Anxiety as a reaction, kind of reactive anxieties from taking street tablets and that kind of thing. So, I'd say that's the biggest category." (Homeless Health Services1)

"So there are a lot of healthcare needs around addiction. Maybe the most obvious being nutritional issues, particularly for those women who had issues around heroin and crack. Many of those women were underweight, so there was kind of a dialogue going on about Ensures [Ensure® Shake is a high energy powdered nutritional supplement for the dietary management of patients with, or at risk of developing, disease-related malnutrition] and nutrition and food and that kind of stuff." (Researcher3)

Comorbidity, physical frailty, and early ageing are common. Many patients have ischemic heart disease or chronic airways disease made worse by smoking heroin or crack cocaine along with comorbidity of injuries caused by injecting.

"A lot of early ageing and multimorbidity." (Hospital1)

"You know, the levels of frailty and pre-frailty in these populations are very high or significantly high." (Researcher1)

"The existence of co-morbidity. A lot of people have chronic airways disease, ischemic heart disease. Chronic Airways disease made much worse by smoking heroine or crack cocaine. So, you see a lot of comorbidity. The comorbidity would be conditions like chronic airways disease, but also the comorbidity of the drug use. Injuries caused by injecting. Venous ulceration or amputations. So, that patient group have a lot of health needs and that's changed even in 10 years." (Homeless Health Services4)

> “Actually, respiratory is huge. I forgot to say that respiratory is a big issue. We see a lot of them for respiratory.” (Homeless Health Services1)

> “There's a lot of COPD and a lot of women talking about their respiratory issue.” (Researcher3)

> “And they also have a lot of respiratory issues because of smoking. Smoking, heroin, smoking crack. Crack cocaine and lung diseases: awful. There's a thing called crack lung, so there are people in their 30s and 40s now requiring oxygen for life because of the damage that's done. So, respiratory.” (Hospital3)

Other presenting issues include musculoskeletal and dermatological conditions, pregnancy, and high rates of depression and self-harm.

> “We see lots of musculoskeletal and dermatology. Things like aches and pains and skin rashes and stuff.” (Homeless Health Services1)

> “A lot of the women I was talking to were pregnant, which is sort of a funny one to label as health need. So there was that issue of navigating that while homeless. What it means to be pregnant in a shelter, pregnant for periods of rough sleeping, pregnant while taking drugs, and - for many of the women - trying to cut down on drugs. That was such a huge shift to make during their pregnancy. So even they really markedly cut down, perhaps they weren't totally abstinent and what that meant mentally for them, as well. Then, for many of those women, having to give birth or be induced pre their uh delivery, due date because of health concerns for them and the baby, and the toll that took on them, and the knowledge that would have an impact on the child as well. So they were sort of physiological, circumstantial, and mental health needs, I would say around pregnancy that popped up a lot.” (Researcher3)

> “The mental health need was just huge. The the anxiety, the depression, the self harm, the depression,...and how that related to the circumstances women were in.” (Researcher3)

CEs working in hospital settings see many patients in homelessness for deteriorating mental health conditions, overdose, orthopaedic surgical issues, or seizure disorder related to injury or substance use.

> “You’ve people who have deteriorating mental health conditions, they present to us - even though we're an acute medical hospital as such - they can come for that. You've people who come in with addiction issues such as withdrawal, overdoses. You have people come in with surgical issues, orthopedic issues, medical conditions. We've a lot of people in homeless services who have seizure disorder and they're always poorly managed because they don't get their appointments.” (Hospital3)

CEs’ patients experience high rates of childhood and adulthood trauma, and/or domestic violence that tend to precede problematic substance use.

> “There'd be very high rates of experiences of childhood and adulthood trauma.” (Hospital1)

> “I've yet to come across a woman who has a substance use issue who has not experienced trauma, domestic violence, exploitive relationship, some form of that. So it's a very, very high

commonality. Both the evidence base and any research that I would have done would indicate that the majority of women commence their substance use or their substance use becomes problematic subsequent to their experience of abuse or domestic violence, rather than the other way around. " (Researcher2)

"Then I did a project in Mountjoy Prison where we interviewed over 300 prisoners and there was a questionnaire but it was about their childhood and what led them to this. And when you see that the majority of them had two, three or more adverse childhood experiences of neglect, abuse, trauma." (Hospital3)

"I think I expected addiction to be a big challenge for the homeless population, lots of complex needs, traumatic experiences, mental health problems, it's just all very complex cases." (Government2)

Priority healthcare needs

Theme 1 – Promote culture that values health equity (level: societal)

To sustainably improve the provision of healthcare to PEH, CEs outlined a need to increase the social value attributed to health equity.

"The priority is not for our guys. Look at the housing crisis, or the homelessness crisis. With time, it's not getting better, it's getting worse. The policymakers, the parties in charge, wider society… we tend to prioritise the most wealthy. We have to start prioritising those most in need." (Addiction Services2)

Currently, health policies are designed and implemented in a cultural context that values tradition and evades risk to the detriment of marginalised populations.

"You have to remember that things have been set up and taught in a very set way for a long, long time and that's with the hospital at the centre and your specialist in the hospital…. It's really about breaking away and changing the mindset around that. As a nation we're not that comfortable with accepting any degree of risk around things, and that's because we're kind of population focused rather than patient focused." (Hospital2)

As a result, despite worsening health inequalities, long-time administrative barriers to healthcare for PEH persist (e.g., appointment systems, proof of address requirements, management of addiction in hospital) (Hospital1 and 3, Addiction Services4, Researcher3).

"There's a lot of mismatches between how the health system is set up and the practicalities of people that we work with lives." (Hospital1)

"Primary care is set up for people with addresses. It's not set up for people in homelessness. Then the current hospital system for people in homelessness doesn't work. It's never their priority, their healthcare. Their priority is where they're going to sleep tonight, where they're going to get their next meal, where they're going to get money for their next week of drugs. So, coming to a busy public hospital and sitting in a waiting room for hours on end… Then if a person in homelessness is for discharge, they'll hand them a prescription for a long list of medications. They're not going to check, 'Have they a medical card? Have they

money to get it?' Unfortunately, due to the business of the hospital, they treat them like everyone else." (Hospital3)

"We're lucky that our clients have access to a GP while they're with us, so, that's not an issue. So they have time. But anyone that's there on the street homeless, finding a GP can be extremely difficult.

Interviewer: Certainly, because would you need sort of a permanent address to be able to...

You need a permanent address.

Interviewer: OK. So yeah, obviously that would be complicated.

And that's a huge, huge block." (Addiction Services4)

"Mismanagement of withdrawal is a key reason that people can't and don't stay in hospital because that experience is so difficult." (Researcher3)

CEs noted unanimously that the current standard of care excludes PEH and encouraged a shift towards proactive, innovative, patient-centred initiatives moving forward.

"Everybody's so invested in terms of the service they provide. Even though it's not working, it's the only thing they know. So that seems to be the culture and the ethos of all of these organizations. There's a complete infantilization in terms of, 'We have this programme. We need more money for it.' So, they go to whoever needs to fund it and say, 'I think it's great', because it's theirs, right? But in terms of having the wider conversation, in terms of how relevant it is, ... It's the 'one size fits all' approach. We have to start changing the way we're thinking about these issues and responses." (Psychotherapist1)

"Some of the problem is getting more of the chronic diseases managed. Things like epilepsy. Chronic obstructive pulmonary disease, even HIV care, because those are things - epilepsy, for example - those are things that really take a lot more long-term management than the HEP-C programme did. But again, I think the deficits are that we need to be able to think outside the box a little bit in how we're delivering that healthcare to patients. Where it works well is where we can bring the management back out to the community so that the focus is taken away from that very standard, traditional route of 'everybody coming up to the hospital to see a specialist for everything'. There's a lot of gaps there and a lot of work to be done on getting that side up because it's really not it,...it'll be new ground. It's not an established thing." (Hospital2)

"It's very interesting because I think despite our background and social policy development, we sometimes perceive ourselves as behind other European countries. But actually, it's my view that we're really good in Ireland at being really innovative and very often at the Community Level. And I think that actually, ironically, comes down to some of the principles and beliefs that emanate from some negative aspects of our past around, say, the Catholic Church and the funding of NGOs and community-based organisations. For instance, we have

a domestic violence service in Tipperary that has been actively accommodating women who are active in their substance use for years now. 7 or 8 years, and is constantly working on an integrated response to substance abuse, trauma, and domestic violence. There's very few examples of that across Europe. We also have lots of other domestic violence services who are responding to the intersection of domestic violence and substance use, whether that extends to accommodating women who are using substances in some circumstances, potentially if their on methadone or stable in their substance use. Ashley House in Coolmine was very innovative for its time and remains very innovative because it has a full childcare service and women can access treatment with children up to - and including - preschool age. It's this kind of creative, person-centred initiative that's working." (Researcher2)

This will require a cultural shift towards understanding that homelessness and addiction stem from systemic inequity rather than personal choice.

"It's not *just* that it's put down to the drugs when it could be something else. Then there's a rhetoric of blame around that. It's like, "Well, we know what happened, that was probably just a drug. And by the way, they made a choice". And that is completely ignorant of actually what happens. I mean, I work with women. So if you're talking about drug use, you're also talking about sexual violence, you're talking about domestic abuse, and this sort of contextual view that's not widely adopted. I mean, so many of my informants were talking about trying drugs for the first time, often in the context of exploitation, when they were children. It's like, well, we don't let children vote, so I don't know why we're talking about rational choice." (Researcher2)

"Unfortunately, it still happens. You know, discrimination is common and it came up with many of my clients, that people were making comments about their smell, about their appearance, about that it's their own fault that they have an ulcer on their leg or they have a clot or they have an amputation. So, I think education to professionals in healthcare and the wider community is really important." (Hospital3)

"People just see homeless drug addicts and think it's their own fault. But if you talk about their backgrounds, trauma from their childhood that leads into this, it gives you more of an understanding. I think the big thing that hit me - because I used to be one of those people, I'll put my hand up and say I worked in the emergency department here when I was a newly qualified nurse and I'd be like, "Oh my God, they're..." Because you spend so much time trying to help them and then they'll just walk out and you're like, "I've just wasted my time". Now I know it's not wasting your time. So, I was that person too, but we never like, growing up, we never got told about where people were coming from." (Hospital3)

"You know soon there will be the opening of a safe injecting a facility. It's *finally* a positive outcome and hopefully it's going to be open by the end of the year. I'm very much looking forward to this. But you know, [*where it's set to be]*, people congregate *big* time there. And it's a big problem in the community. Because for us as workers, it's fine, we can deal with this. But let's say, you know, there's a school not far. Then there's tension there. And the fact that having this safe injecting facility then I think we will improve will help there. It certainly will help the service user in actually doing it properly and safely. It will save lives, undoubtedly. But until we help local residents see its value, it's going to be a problem." (Addiction Services2)

Theme 2 – Accelerate action in addressing health inequalities (level: policy)

Sub-theme 2.1 –Strategic planning

CEs noted an overreliance on reactive, shortsighted healthcare strategies implemented in response to co-occurring housing, addiction, and mental health crises.

> "It's just not thought out. The government will come out with...and again I use that 'reactive policy' because that's what it is. They'll say, 'Ok, we've drug deaths at the moment, how are we going to deal with that? Safe injecting rooms? Ok, we'll do that.' And everybody jumps on the bandwagon and it's a big fuss about who gets the funding. The funding's given and nothing happens. That's the same with community detoxes. All that kind of stuff. Suboxone would be another one." (Addiction Services1)

Instead, they hoped to see policymakers take the lead in developing a wider and more strategic approach to achieving equitable health.

> "15 years I'm doing this now and there's been billions pumped into homeless and addiction services. I think there's about 55,000 people now working in the industry and yet, it's getting worse. It's always reactive policies. It's consistency we need and actually talking to people and thinking through long-term strategies." (Addiction Services1)

This will require the redistribution of resources in proportion to relevant needs and long-term funding allocation for innovative but evidence-based initiatives.

> "The priority is not for our guys. Look at the housing crisis, or the homelessness crisis. With time, it's not getting better, it's getting worse. The policymakers, the parties in charge, wider society… we tend to prioritise the most wealthy. We have to start prioritising those most in need." (Addiction Services2)

> "What we wanted to achieve as well is to have more of a shift over to evidence-based care. So just to make sure whatever funding we have invested in homelessness, it's actually more so evidence based. Because sometimes, you know, good intention does not equal good intervention." (Government1)

> "Well, I think if it's related to project development, quite a lot of it can be related to funding. You know, in order to kind of make sure that the project is sustainable in the future, you need to have reoccurring funding or dedicated funding to it, meaning that you're going to have funding each year for that specific project. And you know, there is a crisis now at the moment, so maybe you might get less funding then maybe the last year and so on. And we can see that the numbers of people entering homelessness are constantly increasing, so the demand is high. We don't always plan enough for this. I think that what we hear from a lot of the regions is that some of them are really struggling with capacity,...their capacity regarding the accommodation they can provide. So maybe funding would be one of the bigger ones because you have so many projects you want to do and you know they can actually have an effect for people as well. It might be just hard to get the funding for them as well. It can be quite competitive in that space, I guess." (Government1)

"It always comes back to funding. It's easy to fund things that are established and we know work. But when you're trying to do something that's a little bit novel or new or outside the standard of care, it's more difficult to prove that works without having funding, but you can't get funding without proving it works. So it's that kind of cycle of things. And I think it's changing a little bit. I mean, people definitely have seen the success of certain programmes that have worked that are kind of more community outreach and the whole goal is to make things more community-based in terms of management of chronic illnesses. So, I think it will get better and there will be more funding available but it's in its infancy. So at the moment that would be the biggest stumbling block." (Hospital2)

Vulnerabilities specific to youth, women, migrants, and ethnic minority individuals in homelessness were a recurring theme throughout interviews, underlining the urgency of strengthening social protection policies for each group.

"Interviewer: Yeah, because I was going to ask you, if there's even subgroups that are coming in that you see have particularly problematic needs and you just said. Elderly with dementia and people facing intellectual disabilities, are there more that come to mind?

Travellers, because it's all that intersection so everything that you have is an extra layer. You know, they kind of pile up. People who are migrants who don't have rights to welfare and housing, so they have no income and theoretically, no right to free medical care. Now they get it, but they don't legally have a right to it. So, all of that. Women, I think it's very, very, very hard to be a homeless woman. There's a lot of sexual violence and, you know, having to go back to the same hostel you were raped in the day before. That's an actual example. A lot of homeless women will stay with a violent partner who offers them protection from other men rather than feeling able to leave that violent partner. And teenagers. So, we would see a lot of people who've come out of foster care. So, you know, when you're 18, the State's duty to you ends and they won't pay for you to be in foster care anymore unless you're in third level education. So the most vulnerable ones aren't and go into homelessness and 18 is very, very young to be in a homeless setting, it's very challenging to navigate anyway. So, 18-year olds coming from foster care. Yeah, that age group tend to do really badly. A lot of the ones that we would have known are dead. You see a lot of early death - people in their 20s, 30s, 40s. It makes me sad." (Hospital1)

"So, I guess one more thing is that quite often people see homelessness as one homogenous group. You know, but there are so many differences, and so many people as well with intersectional identities. You know, women might have specific needs and then as well, there's a piece of work we would like to progress in relation to sex workers and as well, you know, those who might be at risk of homelessness or in homelessness and engaging in sex work. Or those from a migrant background, for example, as well the LGBTI population, they might be at high risk homelessness. So, it's really kind of like, it's not just one group of people. You know, the experiences within the group can be quite different and that requires a different set of policies and interventions to set in place in the future. It's not a one-size-fits-all approach." (Government 1)

" I'd also say that a lot of my informants were Travellers and they had had *terrible* experiences and a lot of women who were Travellers would say, "I'm not going to ED because they're just

gonna treat me like a 'insert slew of sort of, uhm, stigmatic derogatory terms against others'. They were like, "If you're a Traveller, they treat you like a Traveller and they don't think that's a good thing. So that was,...that came up again and again and again, and the hurt around that. I guess in terms of that word stigma, like what are the stigmata of being a certain ethnicity that you can't do anything about and elicits a certain reaction from somebody else?" (Researcher3)

"I had one guy who broke both his hips and never came into hospital, like walked around on a broken hip at home. Can you imagine the pain? We only found out about it years later when he was in for something else. And he's a Traveller who's homeless since he's 11, who has alcohol dependence. So, you can really see those layers building up there." (Hospital1)

Sub-theme 2.2 – Housing First

CEs emphasised that efforts to improve someone's health cannot work if that individual does not have safe and stable housing.

"...that barrier of, 'I'm living on the street and I don't have access to a shower or clean clothes and now I have to walk into a place that feels very daunting and I've to sit in the waiting room with other people, and I've just sit there on my own because maybe I don't have family contacts or friends that could come with me'" (Homeless Health Services1)

"So I think what's really important is going forward that there should be more housing available for people in addiction because, I mean, how can you expect somebody to get off drugs who's living on the street? You know, they go to a hostel and there's lots of people using drugs around them. So you can't be expecting people to stop. So that'd be my thing. That in order to get people to move on from their addiction, that there needs to be housing available for people and *then* we look at their addiction. You know people need stable housing in order to focus on the addiction." (Addiction Services4)

Dissatisfaction with temporary accommodation for PEH in Dublin was mentioned by all CEs. Homeless hostels are often detrimental to health due to ubiquitous use of drugs, physical and sexual violence, noise, and lack of storage for medications (Addiction Services4, Researcher2, Hospital1, 2 and 3).

"You know, they go to a hostel and there's lots of people using drugs around them." (Addiction Services4)

"Women, I think it's very, very, very hard to be a homeless woman. There's a lot of sexual violence and, you know, having to go back to the same hostel you were raped in the day before. That's an actual example." (Hospital1)

"If you're homeless and you need insulin, where are you going to store it? You need a fridge." (Hospital3)

"Interviewer: And was it possible for people to [avoid drugs] and still get the care that they wanted or needed?

Really good question. It's so hard because people were sort of able to do it at a primary care level. People were able to be like, you know, they were saying, "Oh, look, you really shouldn't be getting your methadone there. Please don't make me go down there. I use more. It doesn't suit me. I like this place much better. But then, you know, were they able to get the housing? Were they going back to a room where people were using?" (Researcher3)

"The housing piece is so central for [people in the early stages of addiction] because the homeless sector,...you know, the hostels and even the long-term accommodation hostels,...There is very, very high levels of drug use and addiction in those and it's very difficult if you're just starting off in that setting not to get involved in, sucked into it. So, that's definitely a frustration." (Hospital2)

"Look, we're in the midst of the housing crisis which is affecting everyone. But the ones who are actually most affected are the most vulnerable people and that certainly has a huge impact. There are services, there's not enough, sometimes they're not optimal especially when clients have to share rooms. There's violence associated with this and stuff." (Addiction Services2)

CEs mentioned clients who preferred prison or sleeping rough to temporary homeless accommodation (Homeless Health Services5, Addiction Services1).

",... a lot of people won't go to the hostel. They sleep on the street because they're afraid to go to the hostels because the hostels are quite dangerous." (Addiction Services1)

"In winter especially, people don't seem to mind prison so much. When I was working in *[prison setting]* I'd have clients get out on a Friday and be back in by Monday. It's sad. People see the conditions as better than in a hostel where you're six people to a room." (Addiction Services5)

Some PEH – especially those without proof of Dublin residency – struggle to access temporary accommodation at all.

"There was some waiving of rules [during Covid] around who could access accommodation that I think have more or less gone back now, in terms of - if you can't prove that you're from Dublin it can be very difficult to access any kind of hostel." (Hospital1)

Some PEH rely on the emergency department (ED) as a safe place to sleep. Others are being discharged from hospital with no place to go.

"From the frequent attenders, the first thing you were saying, I would say there are two groups of people: there are people who will return frequently because of some recurrent medical need…Then there are another group which is the other half who don't want to sleep in a homeless hostel because of all those problems I said. And they literally show up for somewhere safe to sleep every night and they're gone in the morning. That group like, outside of appropriate kind of safe housing, there is nothing that we can do for that group until we

have that. It wouldn't even have to be that they get their own house each, or whatever, but some kind of safe accommodation. A lot of them won't have addiction issues. You know, they might drink alcohol, but they won't generally have any,... opiate addiction issues is probably the best way to say it. But yeah, so they just wouldn't feel safe and generally they're a little bit older and things like. They just wouldn't feel safe in homeless accommodation so they use the ED as that kind of support." (Hospital2)

Four CEs mentioned the effectiveness of the Housing First programme which, by providing permanent housing for people experiencing homelessness without any preconditions around addiction or mental health treatment, views housing as healthcare (Government1 and 2, Addiction Services1 and 4). CEs hoped to see an expansion of this programme (Government1 and 2, Addiction Services4) accompanied by more extensive wraparound supports that include daily living skills training (Addiction Services1).

"Interviewer: Of course, of course. And have you seen? Because I know that now there's more of a focus on the housing first initiative. Have you seen positive results of that?

We have seen positives, but the demand for that housing is so high that there needs to be more." (Addiction Services4)

"I read the evaluation of the Housing First programme. And that was very eye opening and most of, if you look at the experiences of the clients themselves, most of them are extremely positive, and yeah, kind of touching in a way as well. So, I think that that's definitely one of the most positive things I've seen and I've heard a lot of positive feedback about Housing First national programme in general." (Government2)

"Housing First is a specific intervention for people experiencing homelessness with highly complex needs and long-term homelessness. So, you kind of just give them a house without any conditions attached to it. So, there is no necessity for them to access treatment before they enter housing. It's really about the choice and delivery of wrap around health supports. And that model has been now scaled up nationally, for example." (Government1)

"I studied Housing First which was a *fantastic* concept. But again, in Ireland, the wraparound services never seem to materialize. So it's just one service doing all of,... which is just not how it works. You need support, *especially* coming from homelessness. Situations where people are going into houses and apartments and they can't cook. One chap I was working with ten years ago who left the oil in the pan, full whack, went in to have a chat with his friend and decided he didn't want chips in the end and he'd watch the football and burnt his apartment down and half the bloody block because he didn't understand that you couldn't do that. He didn't know how to put on a washing machine. He used to sleep on the floor because the bed was too soft. There's just no kind of coordination with it. This chap was entrenched, as like,... a lot of people won't go to the hostel. They sleep on the street because they're afraid to go to the hostels because the hostels are quite dangerous. So you can't just pick somebody up like that and put them into an apartment and then leave them there. In Australia you've got the wraparound, you've got people coming into them everyday. You've got psychiatrists, you've got everything, social workers... It's complete wraparound for the first year. They spot the gaps.

But [laughs] that doesn't happen here. So they can be running around saying, "Oh, we housed 5000 people." But if you actually look at it in the longterm. How many of those people are still in there? Or are they back in homelessness? Because they didn't get the support so now they're back in homeless services. Some of them would have had two apartments and now they've been black balled by Dublin City Council because, "Oh, we gave them two!" But they didn't give them two, they put them into two. It's a completely different thing. So, it's that... I would love to see that happening. When people say, 'interagency wraparound' that they actually know what they're talking about." (Addiction Services1)

Sub-theme 2.3 – Social support options

CEs felt that Ireland relies on an overmedicalized response to homelessness and its associated conditions that fails to address underlying causes such as loneliness, neglect, isolation, and grief.

"A lot of health interventions, particularly for homeless people in hospital, are medication but a lot of the issues are symptoms. They're symptomatic of loneliness, loss, trauma, or whatever it is that life has thrown at them. Or the 'lack'. For a lot of people on the streets, there would have been a lack in terms of mothering, parenting..." (Psychotherapist1)

"Our understanding of these issues is too medicalized. You know and that's part of, like, I really have sympathy for the doctors in that as well because it *has* to be medicalized because the only people responding are the doctors...that's a little bit of an exaggeration. I just mean... Look. If you show up to a hospital, you're probably going to get cared for in some capacity, and I don't just mean that for homeless people. I just mean that, you know, I mean that if you're elderly. I mean that if you're... I mean a hospital is by its nature, by its ethos, a responsive place. But in order to do that, it has to transform the thing you're responding to into medical language. So, the classic example of this, not to bore you, is that elderly people show up because they're old and their family can't care for them anymore and it's a really sad situation. Then the hospital has to try to transform that into a medical issue, and it's not. It's *kind* of a medical issue, but everything's kind of a medical issue. That happens with homelessness as well. We have to medicalize these things in order to - in some way - respond. And we can do that. But in order to do that, we lose all the other things that are necessary. So even, there's this, you know, like as as much as I'm saying, 'No, no. You do have to respond to the epilepsy that might be there.' There's another side to this, which is like, well the person needs stable accommodation. They need a supportive environment. So, you know, you could flip it all around and say there is an issue with the way we're talking about care here in terms of access to medical care and. It's not good to pathologize the issue. Even the way that women were talking about the loss of their child in terms of depression - don't get me wrong, I'm not qualifying that, I'm not questioning that, I agree with that and we're biochemical beings in some way, or physiological, so of course that translates into that - and yet it is *weird* to respond to grief or trauma with a pharmaceutical. Or to reduce it to that." (Researcher2)

With limited avenues for social and emotional support, clients in homelessness often lack the self-esteem required for recovery.

"I suppose part of it is we know that if you have a stable accommodation, money coming in, family support…they're all things that will improve health. Without those, where do you start?" (Hospital3)

"I think there's a sense of not being entitled to anything better. Often, a lot of the people we work with say, 'Oh, I've brought this on myself and I'm so ashamed and I did this to myself.' Because a lot of preventative health, you're investing in your future self, and I'm not sure that many of them maybe have that same sense of a future self that they're looking after. Which is really sad. And then I suppose what we try and do in our work is kind of use other things to substitute for that. So, if they come to clinic, I'll make them a cup of tea and pamper them a little bit just, you know, like if that's why they come to clinic that's fine! It doesn't matter to me because they may not come for themselves." (Hospital1)

CEs considered ways to reformulate how the system views, responds to, and prevents intersecting homelessness, addiction, mental health, and physical health impairments. Suggestions included having a societal conversation on the drivers of homelessness and addiction;

"For me, the big question has to do with, "how come we have created a society where addiction is so prevalent and has become so normalized?" We're always setting up new projects, new projects, new projects, to deal with the symptoms immediately rather than address the causes.

Interviewer: One of the causes being loneliness?

Oh yeah.

Interviewer: And trauma?

Oh totally. Absolutely. And neglect and all of that sort of stuff.

Interviewer: So, what would be better ways that we could address those instead of the symptoms of those?

I think first of all is to have a conversation. Have a societal conversation in terms of posing the question, "how come there's so much violence? how come there's so much addiction? how come there's so much mental health issues? Homelessness?" But these are the conversations that I don't see and that's what attracts me. I wish I had the capacity to sort of really generate those conversations." (Psychotherapist1)

focusing on the strengths rather than weaknesses unique to PEH including resilience, creative problem solving, honesty, humour, and altruism (Psychotherapist1, Hospital1);

"A couple of years ago I did a seminar on psychosis that was kind of hairy because in the room it was mainly people whose family members suffered from psychosis or there was a couple of people in the room who being actively psychotic as well. Right? Which is always a little hairy when you have people in the room who are psychotic... So it went backwards and forwards and then this guy at the front of the room stood up and he turned around and he looked up and you could see from the way his body was moving that he had been medicated for a long time. He was quite rational...and he, first of all outlined his multiple diagnosis, and he had a lot of diagnosis. He said, off and on, he's spent 10 years in psychiatric hospitals and then he

looked at us and said, "The way I see it, is this: when you begin to feed what's right, what isn't begins to fade away." Which is a whole different approach in terms of psychosis or really any type of suffering. You know? It's a whole different approach, it's about feeding what's right rather than addressing what isn't right. So, it's a whole lens through which to see things.” (Psychotherapist1)

“Interviewer: What would be community strengths or factors of resilience within this population that we can focus on?

Oh yeah, there's loads of those! They're *so* resilient. I would be so long dead if I had to do a 10th of what they do so. They tend to be really community oriented. There's actually a huge sense of looking out for each other and minding each other. So, I'll have patients be like, ‘Oh, I saw this lady, she needs to see you. Will we go and see her?’ Or they'll be bringing each other in, or helping each other. When they're admitted to the hospital, I'd often notice it's our socially excluded patients, so notice that there's an older person with dementia who maybe is forgetting to eat, and they'll remind them to eat or take care of them. So, there is a real sense of community and altruism. I think that everybody wants to help somebody else and it's really important, if you give people who are homeless a chance to help somebody else, they'll usually jump at it because they really want to give back and help other people. So that's one. I think they're, there's also a lot of creative problem solving. Like, literally, how would I survive with no money, nowhere to stay and nothing to eat? They can be really good at figuring out how to how to manage something. I think there's a lot of honesty. A huge amount of honesty because there isn't kind of space for bullshitting and egos and like, "Oh, I'm posher than you are." So, it's a lovely group to work with because you can be really honest and enjoy that. There's a lot [laughs] of joking and messing around and singing and being silly that I really love. I can't remember - I've worked now with this community for so long - but I don't think it was there to the same extent. There's a kind of a freedom, you know? It's like, well, you know, society thinks we're crazy anyway,... And there can be a real tolerance of other minorities or other excluded groups. There's an openness, I think, that's really nice. “ (Hospital1)

and – in more concrete terms – prioritising funding for community development initiatives.

“The health service, if you're talking about physical health, hospitals, and stuff like that, it's quite medicalised and they deal with people as individuals. I'm very much a social worker that [believes in] community social work. I'd like to see more about that collaboration pieces with ourselves and community development workers, community development programmes, that kind of brings us very much back into the communities. As things stand, we tend to parachute in.” (Social Care1)

“Like the cannabis programme, that was funded for two years, that was really successful. Working with 18 to 25s, young people, cannabis being their starter drug and we also incorporated Mental Health Matters into that piece as well. That helped as well, gyms, walking, sports,...and they loved it. A lot of people - a huge amount of people - had good outcomes where people actually stopped smoking.” (Addiction Services1)

CEs hoped to see more spaces and opportunities for PEH to make social connections (e.g., adult education, exercise) (Addiction Services1, Researcher1).

"One of the huge issues in addiction and in homelessness is loneliness. So, to create some type of forum, place, a venue where people could come along and have conversations.... I would say just looking at AA. AA is so successful because it's conversations between people who've navigated the same landscape. So, you feel known, you feel understood. Very sort of basic things and opportunities to come together. I had this idea - I need a venue for it. There's a concept in Irish, in Gaelic, called Meitheal. Have you come across it? It's this idea of people coming together to help each other. Normally it would be around harvest time, in old Irish time in rural Ireland. So surrounding farmers would come together and all work together to bring in a harvest and then the only payment would be a big meal and maybe some alcohol afterwards. So, there was that sense of togetherness and working together. So, the proposal that I was trying to put together for somebody to give me a room, some place in the city, and it was specifically for asylum seekers and refugees who don't have places to go outside of Direct Provision. During the day they could come together, have conversations, maybe play some games, that type of thing. IT would be an opportunity for Irish people to offer this to people who are non-Irish so there would be a mutual benefit. So that type of thing is very simple and for me, would have a big effect." (Psychotherapist1)

"I would say, from the previous work I did, the older people returned, they came back more [*for exercise classes*], they had more health conditions, they were more frail. So, they saw the value of doing the exercises and feeling better. But I would say part of that was that they weren't in addiction and homelessness was more of an issue. The females, they just loved it. There was a mix of ages with the women. Some of the interventions were one-to-one and the intervention where I'm currently at is in a group setting. But again, the women engage more, the women enjoy it more. It's because it's sociable as well. Definitely, yeah, women and older people have definitely come back more. But I still think the problem comes back to addiction. You know those who are in unstable addiction just can't get beyond that. I think that's the biggest challenge I've had and motivation has been really part of that but addiction trumps it. I would have a group of ladies that come from a Traveller community and they come together and they love it. They come once a week and say it really helps with stress. You know, the fun of it and the social aspect of it, which is great. We've also, so we do 2 exercise classes a week and we do a park walk on a Friday, so we walk in a green space. Just a self-paced walk. Some people choose that over the exercise class. Our overall aim was to provide an opportunity to exercise three times a week considering WHO physical activity guidelines, just a target to exercise three times a week. People who have difficulty engaging would do the walk, you know, the walk and the talk, more easily. It's powerful, it's free and brings people together. It's such a good resource." (Researcher1)

For individuals for whom addiction impedes participation in social programmes, showing patience, compassion, and advocacy in the provision of healthcare was mentioned as a way to encourage self-confidence (Researcher1, Hospital3).

"There's people and I have been 3-4 years chasing them to come to an appointment, but you just keep trying. And when they're ready, it *will* happen, mostly. So, it's about giving them time. Feedback as well was that nobody ever cared enough about them to chase them as much as I did. They said it's the first time in their life they felt like they mattered. So, those little things, you don't get that all the time, but it's nice to know that, you know, you can make

some sort of a difference. And to hear that, to say that nobody ever cared before to put so much time into them is sort of sad really.” (Hospital3)

“So, one of the mobile clinics has peer support, so you've got people who were homeless before or who were in addiction, and now their job is peer support. So, they kind of initially, most of their job would have been if somebody has a hospital appointment, can you accompany them to the hospital appointment and, you know, give them a bit of confidence,...that barrier of, "I'm living on the street and I don't have access to a shower or clean clothes and now I have to walk into a place that feels very daunting and I've to sit in the waiting room with other people, and I've just sit there on my own because maybe I don't have family contacts or friends that could come with me"... So that was the initial idea with peer support, to accompany people to appointments in the hospital.” (Homeless Health Services1)

“We're all about just trying to do whatever the person needs and being non-judgmental. Trying to be nice, basically. I’m thinking of one nurse… She just really has a way of making people feel safe and cared for that then enables her to deliver all kinds of therapeutic interventions that other people couldn't.” (Hospital1)

Sub-theme 2.4 – Interagency collaboration

CEs reiterated, as has been widely documented, that homeless individuals have long standing and sometimes chronic health issues which relate not just to housing but to a conglomeration of issues such as poverty, substance use, domestic violence, tragic accidents, and social isolation. Despite the intersection of issues, Researcher2 noted that historically in Ireland, substance use has been viewed and responded to separately from health.

“In terms of health, long standing and sometimes chronic health issues may not be completely related to substance use. It’s related to poverty, housing, potentially domestic violence, tragic accidents, incidents… a conglomeration of issues that require additional support and back up.” (Researcher2)

“The other thing I think is historically, when you look at drug policy and intervention, you know it was allowed to (1) develop through a voluntary or an NGO kind of pathway. It went - for a period of time - under the umbrella of mental health and psychiatric services, and there was some private response. But I suppose substance misuse has traditionally been viewed as an individualized issue or problem. It was never viewed historically in relation to its connection to other issues. So, I think it gets difficult then to ensure that the system responds to substance misuse and all its related issues.” (Researcher2)

Today, according to CEs, homelessness, mental health, and addiction services continue to act as separate agencies with little communication between them.

“I mean you’ve got homeless services, mental health, and addiction all acting as separate agencies. We don’t talk to each other. Well, actually, mental health can refer into our service, but we can’t refer to them. It all goes one way. It doesn’t have to be like that. I’m just thinking of clients I’ve had with private health insurance… for them it works beautifully. Health care, mental health and addiction sit around the table. Everybody knows each other and communicates because the money is there.” (Addiction Services3)

> "So, I think maybe the biggest gap and the biggest challenge that we need to address going forward is more so a shift over to integrated care and joint cooperation between services and that would tie back into data sharing policies as well." (Government1)

Many services (e.g., probation, housing, addiction, healthcare, mental health, child services) are involved in one care plan and communication is not always optimal.

> "Very often service users have multiple key workers. Let's say people in hostels, for example, and in hostels in STAs, they have a key worker assigned to them, but the key is really sometimes the communication between different key workers is not kind of optimal, you know." (Addiction Services2)

> "So, for example, if you think about a person who might have been in State Care in the past, they will be under the care of Tusla. After they turn 18, they have to leave that care and sometimes it can happen, quite often they would actually enter homelessness, but they might still have their aftercare worker from Tusla engaging with them. Then, they might have a case manager with an NGO. They might be linking maybe as well with healthcare, for example. So, you might have different service providers involved in a care plan." (Government1)

To address this, truly comprehensive case management and routine interagency meetings were recommended moving forward.

> "It's just a matter of kind of really centralized case management having a good conversation. Let's say you have a client that has also a doctor, a probation officer and then key workers in hostels, key workers in the community, in an NGO, and it's just ensuring basically that the communication between all the services is effective. So that's what case management is about. A big part of what we're doing at the moment, at least in Dublin, is providing case management where people live in hostels and that seems to be working." (Addiction Services2)

> "Case managers have to be involved in every single aspect of someone's care. Housing, mental health, addiction, life skills, all of it. There has to be a link between all those agencies" (Addiction Services4)

> "But the health service, if you're talking about physical health, hospitals and stuff like that, it's quite medicalized and they deal with people as individuals. Where I'd work with them as members of a family, that family really inserted into a community, and how the community and the services there, how they support that family. So, we operate under what's called an ecological model. So when we're working with children, or families, we look at all the different areas: mental health, physical, health, finances, school. We look at community, we look at housing, we look at all the different areas. They're called Meitheal Meetings. It's an old Irish name. It's social capital, people coming together, working with people in all different disciplines. How we have a family here. Here's what the needs are. Here's where we need to get to to see that the needs are being met, and how do we work together to get from A to B? I do them meetings - they're multidisciplinary meetings - so ourselves, hospitals, mental health, housing, and department of social protection. All them areas come in to play. So what we do is, we tend to have a child, but more often than not, you can't really untangle the needs of a child from the needs of a parent because they're so interwoven. So, yeah, it is community development. So crèshes, schools. Mom could be, let's say, on a drug treatment programme

at the Dales in Darndale [addiction treatment centre]. She could be attending a counselling service there, and they'd come in on board. And our young lad or our teenager or our daughter could be missing lots of school and more often than not they have all the D's and the O's as I call them: the ADHD, the ODD. These things cluster together. Mom is working on her recovery trying to get her head back in the game, if you like. Trying to work on herself, and all these other things are presenting as well. So rather than just coming in and working on one area, we look at the whole,...What are all the areas that impact this family? That impact this young person? That impact this parent? and how can we work with all them areas to help? But it's very much interagency. It's very much coordinating the plan. It's very person-centred. It's very solution focused, but it puts the parent, the family, at the centre of it. And it's *their* plan, how they see their lives. And they look at a child and a family, not just in one area, they look at them at all the different areas in their lives." (Social Care1)

"If you think about it, clients don't know what's happening with support services when they're dealing with them all in different silos. When they all come together and actually have a meeting, they're much more informed. All the other services are also much more informed. They're seeing the person in a much broader perspective." (Social Care1)

Sub-theme 2.5 – Data sharing & linkage

CEs noted that lack of data linkage between agencies contributes to the compartmentalisation of care.

"So, I wouldn't say that there is a lack of data on homelessness. I think there is a lot of data on homelessness. A lot of NGOs would be, for example, doing their own assessments of people when they're just entering the services and quite often those assessments would be holistic in their nature. So, they would be assessing housing needs, health needs, maybe employment and training and so on. Then, you have homeless people interacting with the wider health system. The problem is that those data are not linked at the moment. That is definitely one of the main gaps, I would say, looking at the way forward. There is definitely a lack of data sharing procedures or policies between different departments, maybe different services, or different NGOs as well. So yeah, I would say that there is actually a massive amount of data in the system. It's just that they're not maybe as easily accessible, or people just do not link them." (Government1)

Separate electronic records systems and lack of individual patient identifiers impeded work, specifically. While homeless medical services use a shared system, that data is not accessible to normal GP practices and vice versa (Homeless Health Services4), nor are primary care records linked to secondary care records (Homeless Health Services5).

"I think at the moment, the homeless medical services aren't integrated enough. You know, the one thing that is good is that the computer system is shared by the homeless services so we can see if somebody,...and that's essential, because if I'm trying to manage addiction and people can present here and then go somewhere else and present somewhere else, it's very problematic, particularly with some of the medication seeking. I can see very clearly where somebody else is has shown up in the homeless health services.

Interviewer: Oh, well, that's very good. And that would be true for someone even if they're going to their normal GP. [She nods her head no]. No, that would be different. OK.

See that's what I mean, I still don't think it's integrated enough." (Homeless Health Services4)

"Right now, James's is only hospital in Ireland with electronic patient record. Our team meets with [*other Dublin homeless primary services*] once every two weeks to talk about specific patients and their needs but we're not meeting with or linking in with the hospitals. We don't have a way of sharing data between our services and the local hospitals." (Homeless Health Services5)

In the hospital setting, lack of electronic patient identifiers contributes to repetition in care as repeat admissions are sometimes started again from scratch.

"That would be the goal, that that would be the dream to have the electronic patient record that follows the patient and makes healthcare seamless. But no…Often people get the wheel recreated, they start an admission again from scratch. They'll get the same investigations done; the same treatment initiated for the original duration…there's a bit of repetition". (Hospital2)

Government1 outlined Health Service Executive Ireland (HSE) Social Inclusion's plan to establish data sharing policies between different partner agencies and to look at linking homelessness administrative data with health data by 2026. CEs noted that accelerating these aims will be important to ensure that health policies are data driven in future.

"So, one of our actions within the strategic framework is actually to start looking into achieving more integrated data systems where we would be able to capture more data about the health of people experiencing homelessness. And then as well, we have an outcomes framework as part of our strategic document and within that outcomes framework, we are saying that collecting certain outcomes and looking into how services are developing requires implementing data sharing policies between different partner agencies. But it is a work in progress though, so it's something we hope to develop by 2026." (Government1)

Sub-theme 2.6 – Auditing

Addiction Services1 and 3 commented on how a lack of objective quality control procedures result in a lack of standardisation and transparency. For example, the HSE and NGOs employ case managers to coordinate a service user's care plan across many services (e.g., housing, health, addiction, social services, justice). Yet, Addiction Services3 had noticed instances where purported case managers held a role similar to a key worker or project worker, only dealing with one aspect of a client's care.

"Case managers should be involved in every single aspect of someone's care. All of it. Now we're seeing people being called case managers but they're actually key workers, maybe they just deal with addiction. There's just no standardisation across the services and I think that's because a lot of the evaluations that take place are self-report. 'Do you provide case management?' Sure, tick yes. There's no real accountability. We hire a private contractor annually to come in and talk to residents and staff and it's great because our service improves every time." (Addiction Services3)

Addiction Services1 noted inconsistencies in reported progressions in housing (i.e., clients are just being moved from one hostel to another) and in drug assessments (i.e., a client's 'problem drug' is noted as heroin when in fact there is polydrug use).

“Oh, listen, I've been at lots of meetings where I've been asked... I was asked to leave at one stage because I questioned stats that they were putting out. There no such thing as having 80%,... I said, "I'm sorry but I just can't sit here and watch you put them up there." It was to do with people coming from addiction into housing, and I said, "No, what you're actually..." and I knew because I was in a lot of the hostels run by that particular organization, "what you're talking about there is that you're moving people from one hostel to another. That's not a progression in housing.” (Addiction Services1)

“Very rarely I've met somebody who was just going to the clinic and *only* taking methadone. I've -- they're few and far between. Maybe ten people. But yet, the policies are in place for that whereas the case management piece in the clinics is actually highlighting the fact that, "no actually, these are polydrug users." Then again it was laziness on behalf of a lot of people. A lot of people doing the assessments would just write heroine. You know, you get an option for six or seven different drugs and then there's an option for which is the problem drug. So, somebody can be taking heroine or taking methadone and it's not a problem. But, the problem drug is benzos because they're walking around like they're zombies.” (Addiction Services1)

In addition to impacting the quality of care, mislabelling can impact clients’ convictions in court as their progress is assessed based on the health and housing supports received (Addiction Services3).

“Case managers should be involved in every single aspect of someone’s care. All of it. Now we’re seeing people being called case managers but they’re actually key workers, maybe they just deal with addiction. Clients end up in court and the judge sees they’ve had case management for a year and it’s not working, but they haven’t. Nobody’s looked after their mental health…” (Addiction Services3)

“So, they can be running around saying, "Oh, we housed 5000 people." But if you actually look at it in the longterm. How many of those people are still in there? Or are they back in homelessness? Because they didn't get the support, so now they're back in homeless services. Some of them would have had two apartments and now they've been black balled by the courts or Dublin City Council because, "Oh, we gave them two!" But they didn't give them two, they put them into two. It's a completely different thing.” (Addiction Services1)

Clients – who respond well to fairness and consistency after growing up in unpredictable environments – can further lose their already tenuous faith in the system (Homeless Health Services1).

“That kind of mistake can really drive home someone’s belief that the system is out to get them.” (Addiction Services3)

“So you can see it in the Dóchas [women's prison] I did a job in the Dóchas. A lot of this cohort are in and out or have been in before, and they actually flourish with such a stable environment and set of rules. So in the Dóchas if you go in, you start - or whatever it is, level 0, there's a name for it - you start on level 0 and then as your behaviour,...so over the first week, quite rapidly initially, there's the opportunity to level up in terms of privileges, but it's so predictable. You know exactly what you need to do to get to the next level, you know what you'll gain, and you know what you would lose if you weren't doing what you're supposed to do. And even if it happens to you, it, they don't really react like it's unfair because they're like, "Oh, okay," and they just kind of start again and they build it up. That sort of stability and predictability, that's

kind of what I aim to have with my children. I think they didn't get that en masse. It feels, it often feels like that sort of predictable environment of, "I'm not giving out, I'm not going mad or giving out, but I do have to go back to daily dispensing because you've gone off the rails". And then they're like, yeah, okay, you know, mostly they're not really starting into you to scream at you about that. They kind of know and don't react particularly badly as long as you maintain fairness and those predictable boundaries. (Homeless Health Services1)

Theme 3 – Remove barriers to access (level: health services)

Sub-theme 3.1 – More services

Though health system flaws are not unique to the homeless health sector, service shortages disproportionately affect PEH who face worse health outcomes and increased barriers to care. A lack of vital health services was noted in the following areas:

- **Mental health services**: Lack of beds for psychiatry was highlighted as a "huge, huge issue" in Ireland (Hospital3, Addiction Services2), as was lack of accessible psychiatrists.

  "There's a lack of beds for psychiatry in Ireland. Lack of services. It's a huge, huge issue and of course, if you're in homelessness,... there's hardly nobody who's in homelessness who doesn't have some sort of a mental health issue. Then it goes along with addiction, depression, you know, needing medication for your...Again, for mental illness you need to take a lot of medication every day. Sometimes once a month for injections, but to try and be linked in somewhere that will do that for you. Yeah, it's tough." (Hospital3)

  "Mental health services. Did I mention that already? So not only having access to the mental health services and but also diagnosis and psychiatrists being available to actually do that work free of charge for people who are homeless or have little means. But huge, access to mental health, again would take months before you have a reference and move on to actually working with the clients." (Addiction Services2)

  The shortages result in that patients struggle to receive mental health diagnoses.

  "Another service we do is dual diagnosis. So, people who have a dual addiction and mental health problem. And what I mean by mental health is not depression or anxiety, like *severe* mental health. Schizophrenia and so on and so forth. So. We have a worker who's actually specialised in that. He goes across all the PEA's and just working with people who have a diagnosis. Sometimes a problem is that diagnosis is a problem. You often can see people are behaving crazy but they don't actually have a diagnosis." (Addiction Services2)

  Patients who are diagnosed but not at immediate risk to themselves or others may not be eligible for care.

  "The interactions with the psychiatry service I was hearing *through* women or through other service providers, but not the psychiatrists themselves. And what I was hearing was that, 'I can't get anywhere because of my addiction. They're just telling me to cut down, they're telling

me to cut down, but I'm depressed and I'm taking more drugs in that context.' So, it's like, it's a complete vicious circle. *Or* women were saying, 'Look, I have a personality disorder, but apparently that's not a Category 1 illness.' So women were really struggling with their mental health and trying to reach out but the services were kind of insufficient and also there was this sort of acknowledgement of like, 'You know, yeah like that's, I'm sure you are sad.'

Interviewer: Yeah, yeah. Did the did the women themselves ever say, like, "I wish they would just...", or did they have solutions of, "If only it was like this, or I need this type of care exactly". Or did they just need help and didn't get it?

So, I think women in general were like, 'Ohh, nobody ever does anything.' There was a sense on the ground. It's like, 'I've raised these issues so much and nobody actually ever *does* anything. Or they make phone calls, but nothing ever happens on the back of those.'" (Researcher2)

For those who don't fit criteria for admission, providers generally advise patients to "go home to rest and be with family" (Hospital3); advice that is inappropriate for someone in homelessness.
"Again, huge issues with mental health services in Ireland, especially for homeless because they don't have an address. All of mental health in Ireland, you might not realise, but it's all about catchment area and if you're not in your area, we don't see you. So what do you do for people in homelessness? It's very complex. Often, you and me, if our mental health was suffering, we mightn't fit the criteria for admission, but at least you have a safe place to go home to. Hopefully have some family or somebody there. Mental health services, psychiatric services, they'll give the same sort of advice to the person who's homeless, but they have nowhere to go. They have nowhere safe, nowhere to feel that they can be safe, so it's very difficult." (Hospital3)

Some GPs are responding to the emergency by prescribing mental health medications without diagnosis.

"A lot of people come to our facility on a load of mental health medications but having no diagnosis and they don't know necessarily what they're on or what for. We have a psychiatrist come here once a week for three hours and help sort through prescriptions and make diagnoses and correct medications. We had two women come through with autism which had been prescribed as a mental health issue. They assumed that they were struggling to socialize, and it was their own fault. They didn't understand the symptoms of their developmental disability or that they even had one." (Addiction Services3)

- **Residential addiction services**: Lack of drug treatment beds was of major concern to CEs who see clients waiting between 3 and 6 months to enter a facility (Addiction Services1,2, and 4, Researcher3).

> "Another battle is lack of treatment centres, meaning residential treatment centres. Also there's a wide range of barriers for the clients to actually access the treatment

centres. They need to be able to provide clean urines and like sometimes you engage, you put the referral in which is nice and easy, and then the clients sometimes need to wait three months, six months, you know. And in the meantime, sure we can do different things. But you know, if the client is doing well but then relapses,...addiction is a real, relapsing condition. So, more often than not clients will relapse, they will take more substances. We're there to support the clients in doing that but it'd be nice to have actually faster kind of access to residential treatment and more. More. I mean, it's a disaster.

Interviewer: Because, it's about six months now?

Again, it depends on the treatment centre. You know, sometimes it could be less than that. It could be 3 months, two months. You know again it's so plan dependent and you know sometimes you manage to kind of bypass certain things because it's a medical priority. For example, a doctor can definitely open doors there. But more often than not the clients will have to fuckin' wait. That's just the nature of the system. It's just, again, shortage of beds. There's just not enough. A lot of people come to Dublin because this is where you find the drugs and then people engage with services in Dublin even though they may not come from from Dublin. So it's just completely saturated. It's completely saturated." (Addiction Services2)

"Easier access to treatment. More beds. At the moment, I think there's 15-16 government bed up at Saint Michaels. They've reduced those beds, there used to be two priority pregnant beds up there, but they've stopped the staff. The rest would be community. So, an investment into residentials where you can actually go, like you don't have to go in there drug free, which is ridiculous. You know, I mean people are out in the community fighting to get drug free and to go into treatment. The whole idea is that when they say they want to do something about this, let's get them into treatment straight away. By treatment I mean residentials working with people, not being on waiting lists for 3-4 months in some places and then a referral to go into another treatment centre you could be waiting another 6 months." (Addiction Services3)

"Beds. There's a shortage of beds for drug treatment. I've seen people doing all these literally weird and wonderful things to cut down, which often weren't successful. And then it's complicated by the fact that addiction is so complicated. So, it's hard to maintain that level of resolve with all the stresses in the world. But at the point at which somebody was like, "I want to get into treatment", it was incredibly difficult. I filled out a drug treatment form for one lady who had dyslexia and she sat beside me in GP surgery and she was like, "I can't really do this. Would you help me?" She never heard a reply. Now that could be for any number of reasons. Maybe it got lost in the post. Maybe we filled it in wrong. Maybe, but that was,... I had an experience of a woman being like, "I want to do something about this" and she didn't get a phone call the next day. She never got a phone call. The fact of the matter is, there are women, and men, who resolve to tackle their addiction and that's not responded to. What we

know about addiction is that you've got to... In terms of access to care, I mean, access to drug treatment is just absolute... and it was made worse during COVID. You know, and it's one thing to to complain about it if, like, there's an abundance of services and it's like, "We're doing all of our bit, people aren't doing theirs". But at the moment, people are voicing their desire to tackle their addiction and it's absolutely, it's like, there's a lot of low-threshold services. There are some abstinence-oriented services, not enough of them. There's a gap in the middle, but all of those are overstretched at the moment." (Researcher3)

"So, there's, there's waiting lists. It's like every treatment addiction service. The demand outweighs the availability of beds, particularly for residential services." (Addiction Services4)

Capacity constraints result in that most drug treatment facilities have clean urine requirements and maximum entry doses for methadone (Addiction Services1, 2 and 4).

"That policy of kind of higher threshold response is that there's actually no availability. But no, it absolutely makes no sense because people want to go to residential treatment because they they want to address their addiction on a site where a programme is 8 to 12 months. They understand, they work on it. While some people may not be ready for it, but it makes absolutely no sense to be required to provide cleaner urine but that's how the system is. Now there's methadone so there's different thresholds. Some services say they'll take clients on 40mls methadone, some others, maybe 50 mls. So, there's variability across different services." (Addiction Services2)

Patients in homelessness struggle to meet these requirements without additional support, yet stabilisation programmes that house people while they come down to make the criteria for other residential services are in short supply. Two stabilisation units now exist in North Dublin, but they are not enough to account for the explosion of crack cocaine use for which "the most effective treatment option is to get somebody into a stabilisation residential admission." (Homeless Health Services4)

"The nature of drugs being misused has changed and crack cocaine has become a really big problem. It's really, really difficult to manage at the moment. There isn't, obviously, things we can do to help get people off crack cocaine, and the really most effective treatment option is to get somebody in for stabilisation. Into a stabilisation residential admission. So that has improved in Dublin 1 in the city centre in that there are now two stabilisation units for people in this area, Barrymore House and a second one in Phibsboro. So that's a change that has happened." (Homeless Health Services4)

"I've had cases like this. They've finished their detox, they're stable around their methadone. They finished their detox over, let's say, six months. Five, six months ago. They no longer use crack, or very sparsely. Then they're ready for it and they can go to residential treatment. So, there's a lot of stuff you can do around treatment in the community. I'm a big, big advocate of that. It's really useful. But for access to residential treatment, the client needs to provide

clean urine. Urine free of any substances, including cannabis. You know what I mean? So, it's really hard for our clients, quite frankly, to be able to do that. You know, there are some services like the stabilisation programme that Peter McVerry one. They can take people who are still in active addiction and help stabilize their drug use. But there's not many of services like that." (Addiction Services2)

"Personally, the main thing that would be great is if there was more stabilization programmes available. I find that there seems to be a huge dip in that. That because we have our criteria, people have to off drugs and have given clean urines, at least three clean urines prior to admission. But I do think that if there was places that people could go to stabilize, just come down to make the criteria for other residential services, that would be amazing because I think that's where we're lacking at the moment: stabilization programs. Particularly for women who are pregnant, methadone stabilization and suboxone stabilization programs would also be amazing for pregnant women because there's so few available. There's hospitals that provide sort of daytime stabilization programs. But I think there needs to be more direct stabilization programs available." (Addiction Services4)

- **Women-centred services**: CEs wanted to see more services that respond to the intersection of substance use, domestic and sexual violence, homelessness, and motherhood. Specifically, there is a shortage of methadone and suboxone stabilisation programmes for pregnant women (Addiction Services1 and 4).

"Stabilization programs. Particularly for women who are pregnant, methadone stabilization and suboxone stabilization programs would also be amazing for pregnant women because there's so few available." (Addiction Services4)

In terms of residential treatment, encouragingly Ireland is one of few countries across Europe with a facility for domestic violence victims active in their substance use, and two more for mothers with children under five (Researcher2). When capacity is reached, however, or for women with older children, recovery remains a challenge.

"So, I suppose a lot of my research is focused on this element because I became very interested, having been involved in setting up a domestic violence service. Well, actually a number of domestic violence services now that I think about it, in different places in Ireland. You know, because I bought into the same thing as everybody else did, which is, you know, the current policy: well, she needs to deal with her substance use before she can access accommodation with the domestic violence service. And, you know, that really kind of brought in this issue or brought forward this issue around that women can't separate out their issues and their experiences. I suppose I was very surprised at the time, even though I was working in the field, at the level of prevalence of substance use with domestic violence. So there are now services and responses within Ireland, but not enough. But that's the same across Europe. Ireland would be seen to be actually leading the field in terms of innovation. So, we now have some women-only services, but again only a handful. You probably know about the ones that exist in Ireland. Coolmine have two residential treatment centres now, and there are a number

of women-only community based and outreach services or women-only clinics and spaces within existing services." (Researcher2)

"Then you have a lack of facilities for women with children. Trying to access services and get child minders, Tusla on their case, it's really difficult. We did a whole report on that as well. I worked with Tusla a couple of years on that one, just, bringing out a report saying, "We need to get them more support". You know, crèches they can go to. At the moment, Ashley House - Coolmine is the only one. They have a facility, and in Kerry now. But very few beds. So that's another huge block for homeless." (Addiction Services1)

"Interviewer: So, in that service the children come with the mothers?

Under fives, as long as they're not going to school, and then at weekends, other children can come. School-going-age children come and visit. There's a rota for school-going-children coming to visit their parents. Parent. And the younger children have a crèche onsite and we can take up to 16 children on site in Dublin. There's a Limerick service and they can take up to 12 in Limerick.

Interviewer: OK. And I would imagine they're all full all the time, is that?

The demand is really, really high." (Addiction Services4)

Gender-specific spaces are also needed in homeless health services. Many homeless women choose to stay with a violent partner who offers them protection from other men and are likely to present to health services with that partner (Hospital1).

"A lot of homeless women will stay with a violent partner who offers them protection from other men rather than feeling able to leave that violent partner. We see a lot of these couples coming in together." (Hospital1)

- **GPs in the Dublin area**: CEs noted that a recent shortage of GP availability is severely impeding access to care (Addiction Services4, Homeless Health Services4, Hospital3). Homeless individuals who have an address are unable to step down from homeless health services to a normal GP practice with capacity to treat longer-term conditions. The system the HSE has in place requires that someone applies to and is refused from 3 GP practices before they are assisted in finding a provider. Hospital3 noted, "for someone in homelessness, how are they going to do that unless somebody does it for them?"

"Interviewer: OK. Those who are motivated to get a GP, are there enough GP's?

[Participant nods head no] No, there is not. So, what we do now is: you can you pick 3GP practices, apply, they'll turn them all down. Then, when you have 3 refusals the HSE have to provide you with a GP then. But you have to go through the process of applying to three different... So, for someone in homeless, how are they going to do that unless somebody does it for them? I don't think *I* could manage it. So you contact three different practices. You document that the three practices that you contacted have refused because they're all full.

Then you go back to,...there is an e-mail that you send it to and they have to assign that person to a GP. But you have to go through that rigmarole first." (Hospital3)

"You find that an awful lot of our clients would not have GP access until they come into our service. So they wouldn't have a GP, so that's even scary in this day and age. Part of our care is that everybody registers with the doctor before they leave and that works really, really good. So then at least we know that our clients have access to a GP. Now the only thing that we've been noticing recently, because there's a shortage of GPs, that that's a real block. The system that the HSE has in place is that if somebody applies to three different GP's that then we contact the HSE and they will find a GP for them.

OK. So, generally in the end someone can find one but it can take a while.

Yes, but it can take a while. It can take a while, but if an individual was out there on their own, it really highlights the fact that the amount of time it takes to find a GP." (Addiction Services4)

"So, I would work very much to try and get people a GP, a regular GP, where they can make an appointment and have their healthcare. The problem is that access to GPs lately has become very difficult. So, the GPs in the Dublin 1 area are full, their waiting lists are full. So, I'm waiting with a couple of patients to try and get them moved on into general practice. Ideally, we would work with homeless people and as soon as people have an address, we would try and get them a regular local, practice in their area. So, for instance I have a girl today who's pregnant and due a baby in two months' time. I really need her to have a regular GP. She's finding the steps here difficult, and she needs to have a GP do the baby's vaccines and the baby's development. We couldn't do any of that. There's a practice in Summerhill that I'd like her to go to. She now has a home, but they're full.

Interviewer: Yes, it's very clear. Of course it makes me think…it seems like a lot of the solutions are more facilities, more staff, more resources. It's not so much that…do you find an acceptance to use? For example, if someone like her, if you were able to get her into a GP, would that be something that she would want?

[Nods head yes]. Completely. She's stabilised her drug use. She has her address. She's suitable for general practice because of her stability, and also because of her address. You know, I can understand why local GPs don't want to see somebody who is in temporary accommodation and likely to be able to head somewhere else, out in the suburbs. But when somebody gets a permanent address in a new apartment in Dublin 01, it would be important for her to move on. She would have an appointment when she goes to see the doctor, she could get the child's needs looked at, her own methadone prescribed. That's what has become a little more difficult in the last few years than it was ten years ago. There did seem to be more capacity when we had somebody doing well. We helped them get an address and they kind of moved on through the system. Now it's harder to get housing and a good address. And it's hard that the GP's in the area are so saturated. It's to do with the population growth, I think. (Homeless Health Services4)

- **Administration support**: Community health providers in Dublin are facing the dual burden of increased administrative work related to wider-system saturation (e.g., needing to apply for 3GPs before the HSE steps in) and navigating the mismatches between traditional referral/appointment systems and homelessness over the phone and via email (Hospital3). Facing overcrowding in their own clinics, homeless health providers require additional administration support in order to maximise time with patients (Hospital3).

  "Interviewer: How do you feel the needs compare to the allocation of resources?

  Yeah, there's a huge mismatch and more and more I've seen recently, like I spent half this morning following up on people who are given... a lot of the clinics since COVID have gone virtual. Respiratory do virtual. The fracture clinic do virtual. So, they're sending out these crazy appointments, "You'll receive a phone call between 9:00 and 5:00 on the 5th, please be available to take this", but you don't have a phone. And I'm like, "Oh, my God, this guy needs surgery", you know? So we're spending a lot of our time is on the phone, on emails. Phone doesn't stop ringing. You could do with a liaison person as well because that's taking us away from actually face to face meetings." (Hospital3)

  "We've no admin support. So, I'm scanning discharges and reading prescriptions. Yeah, you could actually expand the team, have double the amount of people we have and still be busy, so yeah." (Hospital3)

- **Services outside of Dublin**: A number of CEs mentioned that shortages of homeless services in rural areas across Ireland are contributing to system pressures in Dublin. CEs currently provide care to homeless clients from all over the country.

  "We are more lucky in Dublin than in the rest of the country, because at least if you're homeless in Dublin, there's GPs who provide care for homelessness. That's not so in the countryside. So you know, in rural Ireland and stuff, you still have people are homeless, but there are no services for them. They're getting there, but not to the extent we have in Dublin. So however bad it is in Dublin, it's worse out of it. So even something like that if you go to more rural Ireland. There's lots of shortfalls there, you know. Or smaller towns in Ireland." (Hospital3)

  "So, for example, CHO4 would have quite good addiction response, as well as CHO3. But then I think where the issues would be those more rural areas. So that would be, for example, Northeast, Northwest, Sligo, Donegal, that would CHO1. So, in those areas that would cover large geographical regions, there's just a lack of services in general. But it's not something that's standard across all of the regions outside of Dublin. I would say that some of them are developing a bit better and some of them will definitely require more investment in the future." (Government1)

  "Look, you know, the healthcare system is difficult to navigate for anybody. It's even more difficult if you don't have an address and perhaps aren't very literate in reading or numbers,

maybe. So, I would say there are lots of barriers to accessing healthcare for people. I mean, we're a little bit,...not spoiled,... but I suppose we're in a privileged position because there are a lot of good services in Dublin. People know about them and they go out and they do a lot of good work. Outside of Dublin, there isn't anything like that. People,...kind of socially isolated groups down the country would be very, very disadvantaged in terms of their access to healthcare. Again, it would come back to that they would have to be incredibly unwell before their access to any type of healthcare." (Hospital2)

One consequence of system pressures is that homeless health providers have limited time with each patient and focus primarily on acute conditions and addiction rather than chronic disease management.

"I'm just too busy doing the addiction stuff to really have a long length of time with patients." (Homeless Health Services4)

Sub-theme 3.2 – Safe services

CEs underlined how safety must be built into health services as well as temporary housing. In addition to safety issues connected to gender-based violence, the complexity of addressing safety issues related to drug debts and drug dealing in proximity to low threshold services was noted by CEs (Hospital1, Homeless Health Services4, Addiction Services1, Researcher3).

"…they're going to the drug dealer across the road…" (Homeless Health Services1)

"People who mightn't use [that low-threshold health service] are people who are trying to stay off drugs because there's a lot of drug use around and that's really difficult to navigate. A lot of women might feel intimidated, people who aren't drug users might find it intimidating, and then there's also often a problem for some of the people we work with that they owe drug debts. Sometimes they can't go to services because they are worried that somebody there will know them and be looking for them to beat them up or kill them." (Hospital1)

"So ,some people would say like, but just to reiterate that I didn't necessarily find a consistent thing of like, "We avoid this service." It was more that people built up a relationship in a place that worked for them or had a certain reason to avoid another place, and that could apply to different places and for different reasons. So, *often* I would hear people say, "I don't like going to X service for my methadone or for my needles. I just find I use more around there.'" (Researcher3)

To address safety issues beyond the control of the health system, CEs commented on the importance of having experienced operations staff as gatekeepers within homeless health services.

"Interviewer: How do you handle the fact that being a low threshold but means that clients may be exposed to others using substances they're trying to cut back on? Do you have ways to try and combat that?

[Shakes his head no]. We can't. We can't. We're not the police or whatever. Again, when the drop in was there, there were the most chaotic situations. People would overdose on site. Drug dealing was happening everywhere, you know? And sometimes the service would say,

> ‘Look, your behaviour is inappropriate. There could be violence.’ So the service user could be banned for week or two.
>
> Likewise at the [*walk-in health clinics*]. It's just, you need staff. You need good staff, experienced staff that are well accustomed to it. They’re used to it and they are respected also. You know likewise sometimes you have violence at that clinic and sometimes people are shouting at you and screaming at you. I guess you need a bit of experience around all that stuff on how to deal with those situations.” (Addiction Services2)

> “But the other thing about access to care that is happening in [*the walk-in* clinic] are the manager/receptionists whereas in the ED you have a bouncer. Immediately, that's connotations of prison...it's completely different. And I'm not suggesting every hospital can have an MC. But there is an understanding around – you know I'm complaining that doctors don't know enough – There's a system in place in *[the walk-in clinic*] to make people feel included and safe and it's not plastered on the wall, and it's a lot about the receptionist, manager staff, and they're doing a lot of access to care work.” (Researcher3)

The provision of training for healthcare staff to spot and report signs of intimidation or violence was also recommended (Addiction Services1).

> “There’s so much intimidation. So, it's basically, a lot of people are building up drug debts and families are being intimidated, all kinds of stuff is going on. We work with superintendents in each area. There's a liaison superintendent. We go out to all the organisations in their area - North Area - and train them up. The credit union, social workers, probation, healthcare, everything so that they can spot the signs and direct them to us. Then we link in with them. It's about training everybody to kind of keep their eye out because it's a *huge* problem at the moment. A huge problem for the homeless, as well. But it's not, you know, they don't really report it.” (Addiction Services1)

Homeless Health Services1 and Researcher3 also hoped to see conversations commence on how to create safe services for people trying to manage their addiction without creating additional barriers to care for people active in their drug use.

> “What starts to happen is, people start to get *just* beyond low threshold, but nowhere near abstinent. They're not abstinent. They don't want to be abstinent. They can't be abstinent. It's very hard to be abstinent. They're not at that low-threshold place and in *that* zone, it's very difficult. Because you are trying to manage your surroundings a little bit, and put yourself in safe situations, and extract yourself from places where you feel at risk. And *that's* difficult. It's difficult for the service providers because they're trying to say, "Look, we're not putting conditions on people. If someone comes in need, we're going to help them. But, I think that's the sort of space that we,... How do you have a tiered option without making it a conditional option? If you know what I mean. I think that's true of homeless accommodation as well. It's like, we want to, you know, some of it is so noble. It's like, "we don't want to put conditions. If somebody comes to the door in need, we're going to treat them.’ How do you start doing this thing of like, "Well, maybe we need to have particular kinds of service, but we don't want services to be exclusive." So that's the sort of tricky space, but it, sorry more briefly to answer your question, yes, I definitely saw that people were avoiding services where they felt like they used more. that didn't suit them as part of an effort to manage their addiction.” (Researcher3)

"Theres a need for a sort of tiered system, where people have access to different types of general health care at different stages in their recovery." (Homeless Health Services1)

Sub-theme 3.3 – Resolved care pathways

CEs highlighted that care pathways in response to 'crisis points' aren't fully resolved. Domestic and sexual violence, for example, often go undetected in hospital (Researcher2 and 3).

> "I think there's an issue around women, how women are treated, and also, this is a maybe slightly different thing, but like,...domestic abuse and violence, which is another thing that isn't picked up in hospital. Again, the poor doctors are like, "Ah here,... what?! Is that our job too?" But people often present to hospitals with injuries - in the context of homelessness and drug use as well - and that isn't picked up, and then women just don't go." (Researcher3)

The current system also fails to assess and support individuals who experience repeat overdose (OD) (Homeless Health Services3 and 5).

> "We see people who have a lot of non-fatal overdoses, but you know you have three or four non-fatal overdoses in a row. And I start thinking, these are people who don't really care whether they live or die. And we've no method, there's no protocol for what you do if someone has repeated overdose. In other words, there's no intervention created. So, I think, there *should* be. We have to start trying to prevent future deaths from overdose" (Homeless Health Services2)

> "Oh in the hostels? I'll see at least one overdose a day. Sometimes two. A few weeks ago, we had an overdose and seizure at the same time. This was two different people. We've one client, not exaggerating, he overdoses every day in his hostel. We know coming to work that *[client's name]* will OD. It gets tricky because obviously we've naloxone for opioids but this guy's taking benzos and naloxone's not enough.
>
> *Interviewer: Is he going to hospital every time?*
>
> No, no not every time but often enough.
>
> *Interviewer: And what happens from there?*
>
> I think he's seen a psych nurse a few times but usually they just send him back to us at the hostel." (Homeless Health Services5)

CEs noted that identifying and supporting patients at these critical junctures could set them on a road to recovery, however this will require overcoming barriers to detection.

> "Oftentimes it's the straw that breaks the camel's back. As you say, they come accommodate, they self-administer, they medicate, all of that type of deal and suddenly something happens and all the coping mechanisms are no longer sufficient. That kind of crisis is a kind of opportunity as well." (Psychotherapist1)

At the patient level, fear of stigma and shame may prevent full disclosure of an event.

> "Trust and building trust with a women throughout the services is a big thing. So, practitioners talk about, for instance, that very often women would present to some of the health clinics

but would present some issues but not others. So, things that are very often not presented or talked about include anything to do with sexual exploitation or sexual health. Or, that what women perceive to be the acceptable sexual health issues may be presented. For instance, you know, pregnancy risk or whatever. But not aspects that may have been connected to transactional sex, rape, sexual exploitation, or risky sexual behaviour. So, that was one thing, that when women presented to specific health clinics within substance misuse services or community services, they very often didn't present all of the issues that may have been affecting them because of stigma and shame and all of that." (Researcher2)

At the provider level, underlying conditions and traumatic events may be put down to addiction and not explored further.

"You know, it is not uncommon for a doctor... Say somebody comes in and they've had a loss of consciousness. It is not uncommon to meet a doctor who will say, "Well, that was just the drugs, why are we treating that?" Now, maybe it was the drugs. If they've hit their head, they still deserve care. Maybe it was epilepsy. You need to do the checks. You can't just assume that just because somebody's also on drugs they haven't also had a seizure. Both of those things could be happening, and by the way, that person still deserves care. It's not *just* that it's put down to the drugs when it could be something else. Then there's a rhetoric of blame around that. It's like, "Well, we know what happened, that was probably just a drug. And by the way, they made a choice". And that is completely ignorant of actually what happens. I mean, I work with women. So if you're talking about drug use, you're also talking about sexual violence, you're talking about domestic abuse, and this sort of contextual view." (Researcher3)

"In school we didn't learn about terms like harm reduction, trauma, methadone. You know? So then when you're confronted with that it's *so* difficult and it takes one person, one senior person who's working off their context, to be like, "Listen, we don't give out methadone in this hospital," or, "You know, look, I know they're having a seizure but that's probably just in relation to the drugs they were taking," for you to be like, "OK, that's, now I understand that's how I deal with that situation". And you go on from there." (Researcher3)

At the system level, staffing shortages and lack of services result in that healthcare providers are expected to detect and respond to conditions with which they are unfamiliar. Overcrowding limits time with patients to build trust, detect a point of crisis, and refer to specialised care and support. Even if the referral stage has been reached, there may be no space available in the appropriate service.

"I think the other thing that's sort of interesting is like, so many of the social services have been stripped back (I'm talking more generally now, not just in terms of homelessness) that doctors become the only point of care for everything and *then* it's like, "Why are we? I didn't come into this business to manage this stuff!" You know what I mean? Like a doctor might be like, "Look, I'm really interested in MS, and now you're asking me to deal with homelessness. I don't really know that..." It's not that I think that's a fair burden. I think the burden we put on doctors is so. darn. high. It's just that, that's the position that we're in and there are huge gaps of understanding there. I was meeting women who were just like, "Why would I go to the hospital because I will be there hours?" and I couldn't honestly say them, "No!" But again, the poor doctors are like, "Ah here,... what?! Is that our job too?" But people often present to

hospitals with injuries - in the context of homelessness and drug use as well - and that isn't picked up, and then women just don't go." (Researcher3)

"Interviewer: Is he going to hospital every time?

No, no not every time but often enough.

Interviewer: And what happens from there?

I think he's seen a psych nurse a few times but usually they just send him back to us at the hostel.

Interviewer: It seems like they'd recognize how often he's coming in though? That that's not working?

Well, I've worked in those EDs as a nurse and I'm not sure we have much time to recognize anything. Those shifts are just crazy. And then I suppose the feeling is where can you send him if all the services are full? He's not really hurting anybody else, only himself… I can see that being the argument of why he wouldn't get a bed. Maybe he doesn't want a bed anyway." (Homeless Health Services5)

Another unresolved care pathway relates to muti-morbidity. CEs found that patients with multiple conditions tend to get lost between health services. Though actions and pathways are improving around dual diagnosis of mental health and addiction issues (Researcher2, Addiction Services1 and 2), CEs noted that they're still not fully resolved (Researcher2 and 3, Addiction Services2) particularly when another layer of complexity is added (e.g., domestic violence, trauma).

"Right now, we have dual diagnosis, which was a huge gap in Ireland. Basically, if you had mental health issues no one would deal with you if you were taking substances, and vice versa. Now that's getting a little bit better." (Addiction Services1)

"Mental health services and drug services have always ping ponged off each other. 'Oh, it's not a drug issue, it's a mental health issue. It's not a mental health issue, it's a drug issue.' All these families that fall between the stools of other services come to us and it becomes a child protection issue, but it's not. It's a disability issue. It's a mental health issue. It's a substance use issue from the parents." (Social Care1)

"Both the evidence base and any research that I would have done would indicate that the majority of women commence their substance use or their substance use becomes problematic subsequent to their experience of abuse or domestic violence, rather than the other way around. I suppose your question is, well, where, where do they go with that? And that's an ongoing issue, particularly when you add mental health difficulties because, you know, we already have a struggle in Ireland around dual diagnosis of mental health and substance use, and where people need to present. Where there's been some kind of actions and pathways around that, it's not fully resolved. And then don't forget that substance misuse services historically were designed, kind of, by men for men. And that blueprint, you know, has still got some shifting to do basically." (Researcher2)

Parents whose conditions fall between the stools of other services can be reverted to child protection (Social Care1, Addiction Services1); a pattern that has grave consequences for mothers who do not present to health services for fear of their child being taken into care (Hospital3, Researcher3).

> "Then again, the big block for a lot of women I worked with when I was in the hostels -- I would have worked mostly with 80 to 25 young women, pregnant, involved in Tusla cases. A lot of them were afraid to say they were pregnant because they were afraid their babies would be taken. They'd be trying to hide it and not accessing medical care. So, we linked in with a hospital and medical social workers, which are completely different from Tusla social workers. You know, and we try and work on the child's guidelines and child protection, but it really scares them. *Especially* homeless, because if you're homeless, amazingly, it is a child protection issue. You know, so yeah, so that would be another big one that came out of that report." (Addiction Services1)
>
> "Mental health services and drug services have always ping ponged off each other. 'Oh, it's not a drug issue, it's a mental health issue. It's not a mental health issue, it's a drug issue.' All these families that fall between the stools of other services come to us and it becomes a child protection issue, but it's not. It's a disability issue. It's a mental health issue. It's a substance use issue from the parents." (Social Care1)
>
> "We way less women than men in Social Inclusion which has to do with parenting, I think. They're afraid they'll get reported and their kids will be taken away." (Hospital3)

For mothers who *do* present, Researcher3 and Homeless Health Services3 spoke on the lack of designated support or pathway for homeless women facing addiction whose children are taken into care at birth.

> "You see the complete truth about this is that it's kind of unclear. It *is* happening and it's not just because,...but I also met women who got an opportunity to go straight into Coolmine, which is the only residential drug rehab place for mums and babies. So, we don't have clear stats on it. The Rotunda is currently doing a project where they try and look at those pathways. And TUSLA doesn't keep track of all the data, so I have, I think, I have a figure for the amount of children that were taken into care under one years of age for year 2020. I think it's around 100, but I'm like, that's like out of all of Ireland.
>
> So it's a small number relative to children in Ireland, and it's small even in relation to children taken into care. It is happening. It's happening for various reasons. It's really tragic. So, I mean in terms of service providers, service providers be like, "This is one of the worst parts of my job". What I would say in relation to that is there's no pathway, there's no set pathway for women once that happens. There's no follow up, that I could find, for women. I mean kind of healthcare, mental health. I'm not really sure what happens to women in those situations.
>
> *Interviewer: It's quite remarkable.*
>
> And that's a good example because then you you're like, "OK, so we have this pathway and you come into your appointment and you..." But a woman's just after losing her baby, your drug use spirals. She's not gonna make that appointment, or she *might* not make that appointment and how do you build a flexible service that's around *care* in the

acknowledgement that sometimes people are in so much pain that they find it difficult to go through the door. What does that level of flexibility look like?

All I can say now is there seems to be a real gap at the point of separation for the mother. And at that point, then the woman just becomes an adult homeless person, unaccompanied by her child, and it's like...but she's a mom and she's going through stuff. You know, what would that service look like? You know, that gosh, that's a really interesting question. I've kind of veered off what you were saying but it changed her relationship to services *profoundly*. All services. And in truth, I did still see people continue to interact with the GP, I guess. So I guess, you know, there's a trust thing happening. You know, there's so much about engaging in care, which is about trust and trust has broken down at the level of the hospital for reasons that are not altogether the hospitals fault." (Researcher3)

"I had a particular issue where I see a lot of women whose children are putting care and - when they're put in care - they just deteriorate really badly because obviously they suffer from losing their children. And often they're losing their children because they're using drugs, and then when they lose their children, they spiral out of control. So, their drug addiction becomes much worse because they're using drugs to manage the trauma." (Homeless Health Services3)

Sub-theme 3.4 – Trauma-informed education & training

A majority of CEs noted that widely cited attitudinal barriers to care persist in mainstream health services. Homeless patients regularly encounter disrespect, discrimination, blame, impatience, and judgement from healthcare providers (Hospital3, Researcher3, Addiction Services4).

"I think we need to get more education. Things like healthcare professionals can speak derogatory to people in homelessness. Unfortunately, it still happens. You know, discrimination in health care is common and it came up in all of my interviews, that people were making comments about their smell, about their appearance, about that it's their own fault that they have an ulcer on their leg or they have a clot or they have an amputation." (Hospital3)

"It's not *just* that it's put down to the drugs when it could be something else. Then there's a rhetoric of blame around that. It's like, "Well, we know what happened, that was probably just a drug. And by the way, they made a choice". And that is completely ignorant of actually what happens." (Researcher3)

"The other thing that my informant talked about in that situation was that like, she was *so* mortified that they were talking about her in those terms. And then she had to sit there and receive care from where she was feeling judged. There was another woman who had an experience in a maternity hospital where they were treating her one way, and then when they saw on her chart that she was on methadone they came up and were making *all* these comments about what she was doing to her child, and all this kind of stuff and. Then you're so vulnerable in this hierarchical position." (Researcher3)

"There's a lot of judgement they feel. So, they have come in with this perception to us that they're being judged already." (Addiction Services4)

Researcher3 noted that insufficient training around addiction perpetuates stigma. In Ireland, general healthcare professionals – despite the inevitability of encountering addiction in practice – receive very little education on its underlying causes.

> “So which is just to say that like I'm not an expert on this on this. It might have changed since then. I can only speak to my experience. We had, in six years of college, two lectures that I remember. I was pretty diligent about going to my lectures, but maybe I missed one. But in other words, not that many about drugs. And they were largely historical renditions of how the heroin epidemic occurred. Nothing about like, you know: How does an addiction emerge? What is its relation to socioeconomic circumstance? To gender? To violence? To age? I mean, so many of my informants were talking about trying drugs for the first time, often in the context of exploitation, when they were children. It's like, well, we don't let children vote, so I don't know why we're talking about rational choice here! But anyway. So I hadn't heard about harm reduction, and I hadn't heard about trauma until I started practicing.
>
> It's interesting because there are some things you can avoid in medicine. You can avoid tonsillitis, or you can avoid, like, the larynx. I don't know *anything* about the larynx. It was never gonna come up in my practise, do you know what I mean? As a doctor, I could avoid the larynx. You *cannot* avoid addiction. It'll come. If you're a GP, if you're working in ED, if you're on the wards, if you're a surgeon, you're going to meet somebody who is experiencing addiction. And we just didn't get any training in it. So there's no... How can you? So you're just relying on that individual being compassionate. Without that background understanding, it's like, "This person is flying off the rails. What on Earth am I meant to do here?"
>
> I think you could broaden that out. How doctors learn about social exclusion and what that does to the interface between patients and doctors. You learn about the conditions as if the conditions are isolated from the experience of conditions in real life. But actually, where your patient comes from, what their experiences have been of care beforehand influence that interface. I would argue that medical students aren't taught about that. Look, there's a lot to get through. I understand. I understand you need to know anaerobic respiration [laughs]. I understand all that. But it's interesting that you can't avoid addiction, and certainly I had a wonderful college experience and it's not to take a dig at my lecturers. Look at this is a societal thing, but for people who were absolutely going to come face to face with substance use, we were not taught about it, and it was only once I had left the medical profession that I started to learn about terms like harm reduction, trauma, methadone. You know? So then when you're confronted with that it's *so* difficult and it takes one person, one senior person who's working off their context, to be like, "Listen, we don't give out methadone in this hospital," or, "You know, look, I know they're having a seizure but that's probably just in relation to the drugs they were taking," for you to be like, "OK, that's, now I understand that's how I deal with that situation". And you go on from there.” (Researcher3)

CEs recommended expanding trauma-informed care training in both university and medical settings, *“We’re trying to discuss getting funding to do trauma-informed care for all staff, from cleaners to secretaries to porters to nurses to doctors. Across the board.” (Hospital3)* The goal of these trainings would be for all professionals interacting with individuals experiencing homelessness and/or addiction to provide care imbedded in an understanding of trauma experiences and how they shape patients’ views of and interactions with healthcare.

“It’s a knowledge of trauma. It's a knowledge of addiction. It's a knowledge of people's responses to services....People come into the doctors and they're so, I mean, they're scared because they're they're...I mean doctors are horrific. Like who wants to get bad news in a doctor's office? Like, it's horrific. And then they've got all this experience of being excluded. Like, literally from people's childhood they have terrible experiences of services and of being massively let down. So yeah, they come into a service and are prepared for this place to them down. When teams work successfully it’s because their practice is deeply imbedded in an understanding of that stuff.” (Researcher3)

Sub-theme 3.5 – Expanding Inclusion Health

CEs recommended expanding hospital Inclusion Health teams (Homeless Health Services 4 and 5, Hospital2 and 3). Two Dublin hospitals now offer dedicated services providing patient-centred, trauma-informed care to individuals experiencing homelessness. To prevent repeat presentations, the teams support patients to attend appointments, to create feasible discharge plans, and to access medications. At the primary-acute care interface, these services are vastly improving continuity of care by linking in with homeless primary health services about specific clients. The services are also set up to manage withdrawal in hospital, meaning patients are less likely to leave early and return to primary care with unresolved issues.

“And then Inclusion Health has been a very positive development. So, Inclusion Health teams in the General Hospital. It's getting much more wrap around services for our patients. Let's say if they go to Saint James's Hospital where there's an Inclusion Health team which try to, as part of the person's discharge care, link in with the services that will manage on the outside. Our patients are hard to manage in the hospitals. They're not good at keeping appointments. They're not great at planning discharge, but that sort of team will work with the local community and they understand the patients better. So those services have really improved. 10 years ago there was nothing like that. You're trying to get somebody with epilepsy or somebody seen... I saw a guy today who has a big knife slash wound and it's caught his nerve and he's got nerve pain. We made several appointments and we even had people meet him to bring him to appointments and he still misses the appointments because he can’t be found. He's sleeping rough. But the inclusion team get that. They completely understand, and they will give someone a cup of tea when they arrive and give them an appointment soon after if they miss one. Whereas if you were dealing with the general hospital, a missed appointment means back down to the end of the list”. (Homeless Health Services4)

“It's great, I can send a text to someone on one of the Inclusion Health teams and know that I can send them my patient and he’ll be seen straight away.” (Homeless Health Services5)

“In hospitals with inclusion health teams, we talk regularly and there's a good flow of communication there so that people who leave unexpectedly,...we'll generally say to them, "Look, this person hasn't completed their treatment. If they show up, this is the story." and if they show up, they'll come back to us and say, "oh, can we get more detail or whatever. And sometimes that will happen for the other hospitals too, but because there isn't such a cohesive inclusion health service in those hospitals, it doesn't work as well as that all the time.” (Hospital2)

“Because in our Inclusion Health team there's ,...I'm a halftime consultant, I have one doctor, a senior registrar under me full time, and we've two nurses. That's our team, you know. That's it. So, it's a small team and we're kind of limited in how much we can do because of that.” (Hospital2)

“I work on an Inclusion Health Team. Obviously, Inclusion Health is supposed to cover migrant health, Roma, Irish Travelling community, LGBTQI+ community. But because in our hospital catchment area our cohort of homelessness is huge. So, we haven’t a capacity to see everybody that should fall under the remit of inclusion. So, our main cohort that we provide care for are living in homeless services. Either homeless on the street, no fixed abode, or living in temporary accommodation, homeless accommodation. Everyone on the team has a different role, but I suppose my role would be supporting people in homelessness to improve their health outcomes, so supporting them to attend appointments, making sure that there is a good discharge plan when they leave the hospital. You know, that they get the information they need, that they have somewhere to go for dressings, for their medication, for support with whatever they need. Because as we know, and as you probably know, the life expectancy of people in homelessness is much earlier than of those of us who are lucky to be housed.

Interviewer: The patients that you see coming in, would they have kind of a specific set of conditions? Or do you get the whole…?

The whole spectrum because we'd see them from the time they present in the emergency department for whatever reason. So, they can present to the emergency department because they have just nowhere to go. Sometimes, unfortunately, you'll have people who can present every day because they've nowhere to go. You’ve people who have deteriorating mental health conditions, they present to us - even though we're an acute medical hospital as such - they can come for that.

You've people who come in with addiction issues such as withdrawal, overdoses. You have people come in with surgical issues, orthopedic issues, medical conditions. We've a lot of people in homeless services who have seizure disorder and they're always poorly managed because they don't get their appointments. Because how do you get an appointment to somebody who doesn't have an address? How do you contact them if they haven't got a phone? You know, so you'll see them turning up over and over again. So our role will be to try and implement something to try and improve that, whatever is needed. Every case is different.” (Hospital3)

“it's quite a new concept, I still go around this hospital and they go,...I was just in intensive care and they've never heard of,... the nurse looking after my patient had never heard of inclusion health even though she works in the hospital with us. I think maybe five years ago there was one doctor who came up, who worked in infectious diseases, saw the shortfalls for people in homelessness and set up the first social inclusion team. Then as time went on, our hospital decided it should embrace this to try and set it up. So we set it up, so it slowly... Cork, they have an inclusion health nurse practitioner in the emergency department. So, they've obviously seen that they have issues. So, and I know that there are other hospitals in Dublin that are trying to get funding. It's all again, a lot of it comes down to funding, money. We could do with double the amount of staff, but there isn't funding there.” (Hospital3)

Sub-theme 3.6 – Outreach programmes & peer support

CEs commented on a need for more outreach integrated care programmes like Ireland's Hepatitis C treatment programme which brought screening, diagnosis, and treatment out into the community. Managing chronic conditions through outreach programmes was of interest as they are difficult to treat in drop-in primary care or traditional hospital settings (Homeless Health Services4, Hospital2 and 3). Moving forward, CEs recommended testing the feasibility, acceptability, and eventual effectiveness of outreach programmes for epilepsy (St James's hospital have an outreach programme for epilepsy but it cannot match the quantity of homeless patients across Dublin with seizure disorder (Hospital2 and 3)), respiratory issues related to smoking crack (Hospital3, Homeless Health Services4), and diabetes (Hospital3). These programmes will have to anticipate and overcome challenges associated with long-term disease management within a transient population.

> "So, really successful things that we've done to see things getting better over time would be the hepatitis C treatment programme. So, we made it as patient-centred as possible. We really did look at how are we going to get this particular group of people to take this medication to treat their hepatitis C. And it really revolved around getting the treatment to them rather than expecting them to keep coming up to a hospital outpatient clinic every couple of weeks. And we really were able to break away from that really traditional, kind of patriarchal view of how medicine should work and we got the treatment out to the patient in the community in a way that really worked for them. And you know, we've had very good success for it. So Austin treated a lot of people down at the Granby through the Shared Care programme. Trinity Court and some of the other drug treatment centres were doing treatment because those are places that people did attend regularly, so they were good hubs for managing it. So, that was a really successful thing that we did. And the findings were that they felt they would never have gotten cured for Hep C If we didn't do the outreach programme.
>
> I suppose it's a very discrete thing in that people have a defined course of treatment and then they're cured and not everything is as easy to manage in some ways as that. So, some of the problem is getting more of the chronic diseases managed. Things like epilepsy. Chronic obstructive pulmonary disease, even HIV care, because those are things - epilepsy, for example - those are things that really take a lot more long-term management than the HEP-C programme did. But again, I think the deficits are that we need to be able to think outside the box a little bit in how we're delivering that healthcare to patients. Where it works well is where we can bring the management back out to the community so that the focus is taken away from that very standard, traditional route of 'everybody coming up to the hospital to see a specialist for everything'. There's a lot of gaps there and a lot of work to be done on getting that side up because it's really not it,...it'll be new ground. It's not an established thing.
>
> There's some outreach in Dublin. Safetynet would be the dominant one, and then James's run a very good epilepsy programme where they do outreach, epilepsy management as well. They actually have a mobile, a bit like the mobile health unit that Safetynet have, where they go out to the community and will see people at their hostel or if they're sleeping rough or wherever they are. So, they do that and then some of the psychiatry services do some outreach as well, but I wouldn't be totally familiar with all of the services.

So I think that there's a lovely piece of work that could be done around the feasibility and acceptability of outreach programmes for people who are homeless or any of the socially vulnerable groups in terms of proving the value of them. I think that would be the first, one of the first steps. I would think that there's a gap in,...like we really don't have any data that looks at how people who are in homelessness, or in socially marginalised groups would like their healthcare? Or would think is the best way for their healthcare to be delivered. So that obviously, we're not just making all the decisions for them. That it has to be in the community, you know, perhaps some of them feel that actually hospital-delivered care is better for them. And then how that would work...what the barriers are to that working and how those could be met? Whether it's peer support or key working, whatever. So, I think there's that initial piece of work that would be really nice to know,...to lead into the second piece of work and that is how successful outreach programmes would be, you know, and that would be setting up a pilot programme and looking at how it worked and being able to feedback on it. And again, it would really need to focus on a few specific areas because you can't cover everything. It would be too fast." (Hospital2)

"Interviewer: I was gonna ask, if there was something else to target. If, you know, we were going to have the big outreach initiative, what would? What would you focus on next?

Oh yeah, yeah.

Interviewer: I know there's so many.

Gosh, yeah, there is so many. I suppose,...more could be done for,... a lot of people in homelessness have seizure disorder and are not linked properly with care. In this hospital, if you have epilepsy, the waiting list to see a specialist is over 12 months. So you're never...If you got an appointment today, you'll put a reminder in your phone to come in April next year to your appointment. When you're in homelessness, that's not going to happen.

So, that's a huge issue. I'm trying to work on it but we don't even have an epilepsy consultant. We've lots of neuro, but St James's hospital have an outreach programme for that, so they go every couple of months out in the van with the neuro team from James's and they go to where the person, the people are that have seizures, so to hostels and stuff. They might have 12 or 13 on their books for one day and go and see them. But it's not enough for all of the patients across Dublin that have seizures. Because so many people who are homeless have head injuries, have alcohol issues, drug issues...The amount of head injuries because they're in involved in altercations, assault. It increases your risk of having seizures, so a huge amount have seizure disorder for whatever reason, be it head injuries, addiction issues.

But the care is really poor, so I think more outreach programmes with buy-in from the neurology teams. We have buy in from part of them, but not,...because, again, they're all so busy, they're overstretched. And then I'll try and sell it. You have to try and sell your soul to people to say that you know that if you go out and see them, for example, it means your own clinics aren't as busy then. Because you're blocking spots for people who won't turn up, whereas you could give it to people who have an address and a phone and they'll turn up.

So you could say that for epilepsy. And they also have a lot of respiratory issues because of smoking. Smoking, heroin, smoking crack. Crack cocaine and lung diseases: awful. There's a thing called crack lung, so there are people in their 30s and 40s now requiring oxygen for life because of the damage that's done. So, respiratory.

Diabetes. If you're homeless and you need insulin, where are you going to store it? You need a fridge. There's a plethora of...and there are homeless clinics scattered around but sometimes they need specialised care. Yeah, I don't know. You could have a...There's so much to do, but it's like, where do you... So you can just start. So by starting like we did with our Hep C,...but you could roll that programme out to lots, but it, you know, it's again money, it all comes down to funding and money and having money for staff and resources. It's not the most resourced part of the healthcare system, unfortunately. But it should improve." (Hospital3)

All CEs who mentioned potential outreach programmes noted the importance of involving peer support workers with lived experience in addiction and homelessness (Hospital2 and 3, Homeless Health Services1-3).

"But you could be, and I think and there's a great,...a lot of people who do manage to get into a course or whatever. But there is a great desire to help, I mean, they've amassed a lot of knowledge and experience. And there is often a desire to use that, and so if you could support that in a programme where you were identifying the right people, then,..I would only love to have more availability of peer support workers. I tax our peer support worker, I'm like, "Do you think you could go and see this lady, bring her to this place and, and also this one, maybe not the same day because you've already got one..," you know? Because it's time consuming, but it's not expensive." (Homeless Health Services1)

"But there is *peers*. We do use peers. Peers are a growing field in the area, which is an interesting area to explore, actually, the use of peers. So, for example, we're using peers for key working people. We're using peers for the Hep C project. Now, they're used much more in the UK and we're developing it, but that's again a form of integrating the community into the provision of services. So it's bringing that expertise on board. Experts by experience, is what often we term them." (Homeless Health Services3)

"And then we have a peer support worker. So, somebody who has lived experience of homelessness, incarceration, addiction and who is now full-time employee in the hospital. He would work with supporting patients to attend appointments. Supporting them to go into rehab. His main role really is to support them to get treatment for Hepatitis C because he's been employed by the national Hepatitis C programme." (Hospital3)

CEs spoke of the potential value in expanding peer support roles to new programmes for both the employee and client. For the peer support worker, the job is an avenue for rebuilding confidence and routine on the other side of addiction. For the client, interacting with someone with shared experiences creates *"an identifiable sort of bond." (Homeless Health Services1).*

"And the other thing is, I think it's actually a great job if you... if you... it's a job. Peer support can be used for that, so you can catch people who are coming out at the other end. Now you have to have a lot of... it's difficult to find the people who are ready for that as well but I think

there's a great deal of self-esteem and return to normality. You know normality is an awful word, return to functional role in society. There's a great self-esteem value and confidence to be got from being the person who can provide peer support. Because you were there and... and also, I mean it... it eliminates a huge amount of the suspicion of judgement. "I know she's not going to judge me because, well, I mean, she's highly unlikely to judge because three years ago she was where I am". So it's easier to take that hand and walk with her to the clinic because she can be like, "oh, when I went in here, you know". There's... there's a... there's an identifiable sort of bond, or there's a... yeah, I mean you can be very clear that you're highly unlikely to be judged by the person who's like "well, I did this". You know, personally. So I think that there's an opportunity for... for those people who have put in a lot of work, but also that still get kind of stuck because it's hard... I think that it's hard at that end of recovery as well where someone's like, "Well, you've got 4 years missing from... where... when did you last work somewhere?" and you're like, "well, shortly before I started heroin.' That's pretty bleak." (Homeless Health Services1)

Theme 4 - Address knowledge gaps

Sub-theme 4.1 - Optimal addiction care and support

Uncertainties linger surrounding appropriate addiction care and support in response to a changing drugs landscape.

"One of my clients died a few months back. It brought up the issue about failure. She was a highly intelligent woman and at one stage in our conversations, she told me that one of the things she might do was a PhD on addiction because she said there's all of these thoughts, answers, and programmes for addiction. But from her point of view, there were far more questions. So, I was trying to encourage her to do this PhD which I thought could have been of tremendous benefit to her." (Psychotherapist1)

"I think as well, because people have been through recovery before, in the drug stabilisation place or the drug treatment place that I go to, the residential places, it's sad in some ways because sometimes you're admitting someone who gives you the right answer to every question, but it's because they've been through a three-month residential place maybe four or five times before. They can give the right answers verbally, but there's something they haven't managed to address psychologically. And those people, there's a little bit of despair in me when I hear those perfect answers, because I'm like, "Well, that is both true and evidently hasn't been helpful for you to know because here we are again". So, and I think in some ways they can sit in the group - you know they do like group therapy, or group discussions and all sorts of things - they can sit in a group and be a really valuable member of that group for everyone else. But there must be something they haven't yet addressed for their own recovery. And you do sometimes see them get through the whole program again, and you're like, "Nope, it's all perfect." There's still a barrier, there's still something that they,...either consciously or unconsciously are not opening. There's a Pandora's box in there somewhere that needs to be addressed but hasn't been addressed. And they can leave with gold stars all over everything, and everyone thinks they're great and they give the right answer. But you're nearly like, "Ahhh," and you can see it, you can see it in some of their answers. EArly on they can give you a history, they can tell you when it first started to go

wrong, it's just so,... it's too perfect. It's all almost learned rather than experienced recovery. But yeah, you can find those people where you think, "Oh I don't have a good feeling that you're going to get here this time because you've learned all of this, and you can tell all of this, but if you really internalized all of those things, why are we back here again?" And I know that circumstantially you'll get people who fall back into addiction for loads of different reasons. But yeah, those people are the ones that I'm just like, "Ahhh, what is going to be different this time?" And how do you help them to reach that?" (Homeless Health Services1)

For example, CEs were divided in their views on methadone. Some viewed it as *"liquid handcuffs"* (Addiction Services1 and 3), while others noted its effectiveness in preventing overdose and relapse (Homeless Health Services1).

"Oh yeah, always. Always. Probably 90% of the people I encounter, and I hate stats but I can safely say that I've done thousands of initial assessments and always the first drug will be cannabis. "Tried it with my friends, bit of a laugh. Great bump." That led...then they went to buy it off someone else and that led to other stuff, "Would you like to try this?" and da da da da. But now it's cocaine and cannabis. Mixing alcohol... So we're running an alcohol and a cocaine programme at the moment because of the demand for it. There's no... nobody runs a heroine programme, or an opiate programme anymore, because they're pointless. They're entrenched in it - God bless them - with the methadone. The liquid handcuffs, as most of my clients call them. It was meant to be preventative but I know people who're on it since the 80s. It's just, yeah... So that's a complete failure. But people will argue that it cuts down on crime [laughs] well it doesn't really...If you want to believe that." (Addiction Services1)

"Clients have to be on 40ml of methadone or less to enter our facility, then they do a complete detox. We've heard from people who want to come in who are on 130, 150mls. The time it takes to come down from that… the doses are just crazy." (Addiction Services3)

" So, we have some people who are on, so, broadly speaking, we have 3 categories of people: [1] People who don't get any benzodiazepines, which is most people, [2] people who are doing a benzo detox, so they're on a particular schedule of reducing doses every two weeks, and then [3] some people who are with us for methadone, so they have to be coming every two weeks anyway to see us for methadone, some of those people will be on a maintenance dose of diazepam. And usually they're long term methadone patients who have tried to detox maybe more than once and just get to a certain stage, maybe around 10 or 15 milligrammes a day, and just below that they fall to pieces. And some of these people since their early teens have been on tablets, so there is an extent to which we kind of see that,...you formed, you formed your whole personhood and your neural pathways and whatever, you know, whatever plasticity you had at the time is now all set and used to a certain amount of benzodiazepine. Your normal - that's your absolute baseline since you were twelve, maybe - has been on a certain amount of diazepam and if you've tried it and they've tried it and you've come to a reality where, actually, if you give them 10 milligrammes a day, they now are stable. They're not going to the drug dealer down the road or across the road and a certain number of people, they do manage to stay on that ten. The problem with it as a technique, the reason you wouldn't do for everyone, is that often if someone's on 10 they just start to to want more and they end up taking more anyway. But, if you can keep them away, if the 10 is enough for them and therefore they're not visiting the local tablet dealer, and therefore they're not buying

more, you know, there's an advantage to that too. But, it's just really funny to me that people will sometimes be dying to get off their methadone, but would be perfectly happy to stay on their diazepam the rest of their lives. And you're like, "Oh, but this is an interesting turn of affairs, you would just stay on that forever even though realistically it's giving you more sedation, it's giving you more 'fuzzy headedness' or whatever". There are other people who will realise that the difference between, you know some people are on suboxone and some people are methadone, and the difference is probably a little bit of mental clarity or sharpness. There are some people who would prefer, they'll try Suboxone and be like, "No, I needed the fuzziness". Often, there's a lot of childhood trauma and there's you know, again, a lot of substance use going back to, I don't know, maybe 9-10-11-12 , very early. They've never been mentally clear and it's distressing to them to be fully mentally clear, that's not something they can stick, they can't do it. So they will happily stay on methadone forever.So yeah, there are definitely people who want to come off it and I'm of the opinion that, as long as someone's aware of their risk of overdose or their risk of relapse that, they should be welcome to try that. So if somebody comes in and they're dead set on going down, I know there are people who'd be like, "You know, no, I'm refusing to do that because I think it's a bad idea". I'm more of the opinion that,...if I can have a conversation where I can be clear that I think this is a bad idea and that they listened to that, and they accept that, that they know I think it's a bad idea but that they still want to go ahead, then they've their reasons.... It's their life decision. Their reasons are more important than my reasons overall. I can't adequately weigh how much importance that has to them, so they want to risk relapse for the chance of coming off it, then that's something they can do and should be able to do. You know? Now you might also say to someone,...I had somebody in today and she's got down to 50 but that took a while and, has a lot going on and I said to her, "Look, you know 50, you could get into somewhere. You could get in to inpatient detox at 50. Would you just stick at the 50 until you got in somewhere, because then at least you have more supervision, you have more support around you for that period of time where it might be higher risk," and she has stuck at 50 for two weeks and is going to think about it." (Homeless Health Services1)

The question of suboxone versus methadone for the treatment of opioid dependence arose, as did the potential for Ireland to expand prescription of buprenorphine prolonged-release injection (Buvidal).

"There are other people who will realise that the difference between, you know some people are on suboxone and some people are methadone, and the difference is probably a little bit of mental clarity or sharpness. There are some people who would prefer, they'll try Suboxone and be like, "No, I needed the fuzziness". Often, there's a lot of childhood trauma and there's you know, again, a lot of substance use going back to, I don't know, maybe 9-10-11-12 , very early. They've never been mentally clear and it's distressing to them to be fully mentally clear, that's not something they can stick, they can't do it. So they will happily stay on methadone forever." (Homeless Health Services1)

"A lot of our clients seem to prefer suboxone but it's only in the last 4, 5 years that it's become available here and that was a real battle." (Homeless Health Services5)

"In a lot of ways we're slow to respond. Other countries are using Buvidal [prolonged-release injection of buprenorphine] but we're not even talking about it.

Interviewer: Why not, do you think?

It's expensive and I suppose they need proof that it's money saving in the long-term. I'm just thinking about the cost of hospitalisations that happens in the meantime…" (Homeless Health Services5)

CEs held differing opinions on benzodiazepine prescribing. Addiction service providers referred back to their sense that GPs are overprescribing for addiction, whereas GPs had a sense that benzodiazepine intoxication has improved since the implementation of a maintenance programme during the Covid-19 pandemic. CEs did not entirely understand why, *"people seem to be dying to finish methadone, but don't want to finish benzodiazepines"* (Homeless Health Services1). Another 'unknown' relates to sleeping difficulties. GPs are unsure of how to respond to clients who strongly prefer to stay on sleeping tablets even after they are no longer effective (Homeless Health Services4).

"The amount of pills some clients are on when the come to us… I don't think they know what half of them are for. Our general sense in here is that doctors are absolutely overprescribing." (Addiction Services5)

"OK, so the problem for us is...There's one other change that's happened lately - and this has happened since COVID - is we are prescribing more benzodiazepines. So, what happened? When all of a sudden COVID happened, before that we did zero benzo prescribing because we really felt we couldn't, ... Well, that was the wisdom at the time. We couldn't manage whether a patient would go to another doctor in pain. Where methadone is very tightly controlled because of a central treatment list, you can't go to another doctor and get methadone, the list is kept nationally.

The same isn't true for benzodiazepines. So, there are much more problems with prescribing them and also you feel that you're just adding into the amount that they're illicit on the streets. So, when COVID happened and I was part of that response, we had to try and keep people who had COVID, or symptoms of Covid who were waiting for the results of tests, from being out and about. We were trying to accommodate them in hostels and so we needed to prescribe some so people weren't withdrawing from benzodiazepines and needing to be out. So, it was liberalized really in that people who had a Benzo problem, we would see that in their urine. We prescribed a small amount daily, a bit like methadone maintenance, benzodiazepine maintenance. So, more sleeping tablets were probably brought in for the same reason. Scientifically, you know, when I start somebody on a sleeping tablet, I see them on those for 15-20 years. Just, they just won't stop. Can't stop taking them.

Now, the literature would say that they stop having this hypnotic effect after a couple of months and still people are psychologically so dependent that if I don't,...I know you can go through periods of sleep and not sleep and everything but if you blame it on not having your tablet, or even just - the effect from stopping the tablets will be a few days of not sleeping - it's a really big problem. It's a particular problem with the hostels next door to a nightclub, as some of them are, or next door to busy streets that are alive at night. That's compounded by the fact that heroine, cocaine really distort your sleep patterns. So for six weeks after somebody has had a smoke of heroin, their sleep is disturbed for six weeks after coming off.

What to do about it? I could sit here and say to somebody, "But they're not working anymore. For two years, three years". "OK, you might have sleep disturbed for a couple of days after stopping them, just because you've taken them away, but no more than that". They won't give them up. I've had patients say, "Well look, now I'm drinking 5 cans to try and get to sleep. I feel so bad that I'm now vulnerable to use drugs again". So, it's very hard to stop them anyway. I don't know what the answer is, but I know the *whole* benzodiazepine scene has changed utterly from pre-COVID in that most of our patients are on Benzodiazepine, five-three times a day now. Or a benzodiazepine detox. and the ones who are on five-three times a day are doing much, much better. That's what I can see. They're not buying handfuls or presenting drug affected. So that is a change as well. See, it's hard to know. It is *really* difficult. I couldn't have believed how many people would be on benzos now, compared to four years ago. But I think all of us feel that actually people are doing really well. And 20 years ago there were doctors who felt - and if you look at the literature in Switzerland and Germany - if somebody fails a benzo detox, and they're back using, it's a reasonable thing to consider them on a maintenance. It's a low dose and picked up daily at the chemist. So, there's international evidence, and there's also anecdotal evidence that people did it 20 years ago in Ireland, but then? The problem for doctors is that they felt very pigeonholed as to,...poorer doctors prescribed them, or less conscientious, and that you could be in trouble with the medical counsel for benzodiazepine, so...You don't start this conversation lightly with people because it's much more nuanced than than people realise. You know it's not 'a good doctor doesn't ever prescribe them and a bad doctor prescribes them'. It's that you actually see the difference. We were absolutely forced to make a change by COVID and that was a very good experiment, because I think if you ask my colleagues what's their *sense* of how we've been doing since then,...it's that we're seeing far less benzo intoxication." (Homeless Health Services1)

"We have patients who have - because of their addiction - sleeping difficulties, - because of their hostel - sleeping difficulties. If you were to prescribe sleeping tablets, they would be on them forever and that is what I know because I'm 20 years doing this, and I cannot get people who are on sleeping tablets off of them. So, for all of us, there's a problem. I don't know what the answer is, but I know the whole benzodiazepine scene has changed utterly from pre-COVID in that most of our patients are on five Benzodiazepine three times a day now, or a benzodiazepine detox. The ones who are on five three times a day are doing much, much better. That's what I can see. They're not buying handfuls or presenting drug affected." (Homeless Health Services4)

Homeless Health Services1 noted a potential gap in beliefs between doctors and those in addiction, *"Maybe we don't actually have the same aim in mind. Would patients seeking stability ultimately like to be drug-free or something else? Are we having honest conversations where patients feel they can share what their treatment goal would be?"*

"I'm fascinated at the gap in knowledge or belief between doctors and those in addiction, and maybe we have different ideas of what would be the best path because we don't actually have the same aim in mind. Are patients seeking stability… would they ultimately like to be drug-free, or something else? Are we having honest conversations with patients in which they feel

they can share what their preference/their treatment goal would be?" (Homeless Health Services1)

Sub-theme 4.2 - Understanding access amongst vulnerable populations

CEs were careful to point out that they don't know about the health of individuals who do not interact with their services. Vulnerable groups whose health needs are not adequately understood include:

- **Undocumented migrants and asylum seekers**: Eight CEs mentioned how little is understood about the health needs of migrants and asylum seekers in Ireland who are experiencing increasing rates of homelessness. Potential barriers to care amongst this population include language barriers and lack of rights to welfare, housing, and medical care. This group will also have specific health needs: "*a lot of them wouldn't have addiction issues or alcohol issues, but they'd have huge trauma from war*" (Hospital3).

  "I'm not sure there is enough information about the health needs of migrant people who are experiencing homelessness. I think those numbers are definitely going to continue to grow over the next years." (Government1)

  "[Asylum seekers] are landing in Ireland, they register at the International Protection Office, and they're told, 'There are no beds'. This is very new now as in the last six weeks to two months. I'd a man who was diabetic and had to tell him to sleep on the street on that freezing cold night. Maybe a month ago, three weeks ago, on a Thursday, I remember. And he kept on asking on his Google Translate code, 'But where am I to sleep?' and I had nothing. We never did that before." (Hospital3)

  "Well, in the last few minutes that I have you, my question is just are, what do you think the the priority research needs are? Do you have kind of burning questions that would be helpful if someone sat down and spent a few years trying to answer?

  Well, I would love to find out about a bit about the people who aren't accessing any service. I know that people, even some of our patients, go home and and I suspect that there is a body of migrants in Ireland who are being treated by their doctors at home. I've heard of people doing phone consultations with their GP abroad and their GP couriering their medication over to them. We had a woman who had a miscarriage the other day, and she and she got on a plane and went home to Romania to kind of complete it, rather than being here. I know that an awful lot of people,... I think that people are inclined to trust, even if they're fleeing a country where they've been badly treated, they're inclined to trust their health service at home more than their health service in the new country. And I think it would be really interesting to find out how to switch that. How can you encourage people to trust and engage with the health services in the country where they are? And another thing would be, well, I think GPs and doctors in general need to learn about how to use interpreters and the importance of interpreters. So, so barriers to GPs using interpreting services, I think, would be an interesting one because apparently years ago, CHO9 used to provide

funding for interpreters for GPs and the GPs never used them and that's a disaster.” (Homeless Health Services2)

“Interviewer: I was going to ask you, if there's even subgroups that are coming in that you see have particularly problematic needs and you just said. Elderly with dementia and people facing intellectual disabilities, are there more that come to mind?

... People who are migrants who don't have rights to welfare and housing, so they have no income and theoretically, no right to free medical care. Now they get it, but they don't legally have a right to it.” (Hospital1)

“Interviewer: Are there specific ethnic groups or maybe age differences,...certain groups that you think really need to be more thought about or anything like that?

So, I kind of had outlined the various different groups. I suppose there are a lot of undocumented migrants living in the city that are hard to find, in some ways, because they're undocumented and they don't really want to be found. Certainly that's a group of people,...and they're not entitled to anything from the State. So you know, they're a very, very vulnerable group. When they do present, they tend to be in a very bad way.
There are a lot of the international protection, or asylum seekers, who have a lot of chronic health needs and are really not getting the access to healthcare that they probably need. I think there's a gap in in us even knowing how much healthcare need there is in that group, to be honest. We just don't even know the scale of it and we're probably not resourced adequately to go out to try and find out what the healthcare need is, or to deal with it. Because in our Inclusion Health team there's ,...I'm a halftime consultant, I have one doctor, a senior registrar under me full time, and we've two nurses. That's our team, you know. That's it. So, it's a small team and we're kind of limited in how much we can do because of that. So, certainly they're a group that I think would really need a dedicated team to kind of go out and find out what the healthcare need is and then decide how it needs to be managed.” (Hospital2)

“I'm not sure that [barriers to care] are as well explored for other groups such as migrants. In other words, looking at barriers....You know, there is a literature, but no one has looked at the health service usage behaviour of other groups such as Roma, such as migrants.” (Homeless Health Services3)

“ Conversations are so rich. I do understand, in terms of the challenges around refugees and asylum seekers is that it's become polarized. Both sides think they're right and they're self-righteous. But there's no conversation around it. There's no deeper-level understanding. Everyone has a point of view about it without actually talking to the people or hearing from those who are experiencing it.” (Psychotherapist1)

"You know, so. But we do that in like, at the moment 34 hostels, so you know the team has really expanded like, yeah. So that's a big, big part of what we're doing. But we

also still have people in hostels who, let's say, I don't know, I don't have stats around, but some people may actually just have been kicked out from their house, you know, and they end up being in hostels. Or migrants or refugees. They don't necessarily have any kind of addiction issue nor mental health issues. They want to work, but they're stuck, you know. So, I suppose our service has kind of evolved in the sense that we can facilitate and help people, whether they have addiction issues or not. In fact, a lot of our clients now do not necessarily have addiction issues." (Addiction Services2)

- **Emerging homelessness**: CEs were concerned about the transition from adolescence to adulthood for teenagers leaving state care. Many are entering directly into homelessness at 18 (Hospital1, Government1), a situation that is challenging to navigate and extremely detrimental to health. With the ongoing housing crisis, some elderly people with dementia are going into homelessness after forgetting to pay their rent (Hospital1). Finally, people with intellectual disability are falling into homelessness because of their intellectual disability. Little is understood about each of these groups' trajectories, health needs, and how they access and interact with services.

  "For me at the moment, the thing that worries me the most are people entering homelessness with intellectual disability or the ones leaving foster care. If you picked one of one of those groups and looked at their trajectories and how the services are set up to access them. I think that if society as a whole was really aware of what's going on, I think that there would be, there could be change in that system." (Hospital1)

  "We're seeing some - with the ongoing housing crisis - we're seeing some elderly people sometimes with dementia going into homelessness because they can't find accommodation. Where they've lost their rental property because they can't remember to pay their rent because they have dementia. And that's a really bad idea. And the disabilities services are another real pinch point where there are a lot of people with intellectual disability who are in homelessness because of their intellectual disability and they really do badly, and they shouldn't be there. It's not fair." (Hospital1)

  "So, for example, if you think about a person who might have been in State Care in the past, they will be under the care of Tusla. After they turn 18, they have to leave that care and sometimes it can happen, quite often they would actually enter homelessness." (Government1)

- **Minority groups**: Five CEs mentioned uncertainties relating to the health usage behaviour and access to care for Irish Travellers and Roma people in homelessness who face increased discrimination due to their ethnicity. Specific, intersecting identities may further shape health needs (Hospital1, Government1). Research gaps mentioned related to rough sleepers, lone parents in homelessness, homeless women engaging in sex work, and members of the LGBTQ+ community (Government1 and 2).

“As well, certain ethnic minorities are at higher risk of homelessness, including Black people and Travellers, so I think there is a lack of research in that field. I'm just thinking in my head as well,...for example, we know some things about homeless families but then I think there is a lack of evidence at the moment about single fathers, for example, in homelessness. As well, I think there is some work needed regarding rough sleepers. I think the last study that was done was maybe in 2011 or something like that.” (Government1)

“And so and I guess one more thing is that quite often people see homelessness as one homogenous group. You know, but there are so many differences, and so many people as well with intersectional identities. You know, women might have specific needs and then as well, there's a piece of work we would like to progress in relation to sex workers and as well, you know, those who might be at risk of homelessness or in homelessness and engaging in sex work. Or those from a migrant background, for example, as well the LGBTI population, they might be at high risk homelessness. So, it's really kind of like, it's not just one group of people. You know, the experiences within the group can be quite different and that requires a different set of interventions to set in place in the future. It's not a one-size-fits-all approach.” (Government1)

“Interviewer: I was going to ask you, if there's even subgroups that are coming in that you see have particularly problematic needs and you just said. Elderly with dementia and people facing intellectual disabilities, are there more that come to mind?

Travellers, because it's all that intersection so everything that you have is an extra layer. You know, they kind of pile up... “ (Hospital1)

“I think the Roma would have very specific health usage behaviour, which would be interesting to explore. And to see, then identify what barriers there are to access to their healthcare, which I think would be different to homeless people.” (Homeless Health Services3)

“I'd also say that a lot of my informants were Travellers and they had had *terrible* experiences and a lot of women who were Travellers would say, "I'm not going to ED because they're just gonna treat me like a 'insert slew of sort of, uhm, stigmatic derogatory terms against others'. They were like, "If you're a Traveller, they treat you like a Traveller and they don't think that's a good thing. So that was,...that came up again and again and again, and the hurt around that. I guess in terms of that word stigma, like what are the stigmata of being a certain ethnicity that you can't do anything about and elicits a certain reaction from somebody else? There's a question about how Travellers in particular are treated in these contexts. That came up for me a lot.” (Researcher3)

Theme 5 – Include homeless service users in health and social service design, planning, and delivery

Policy and system-level flaws result in a lack of immediacy of care which *“is key when you’re dealing with individuals that have a large degree of instability in their lives.”* (Researcher2) In turn, many clients have lost faith in and disengaged from the system.

> “The other thing: there was a number of examples of women disclosing or,...trust being built up, and the woman getting to the point of seeking support for a particular aspect, be it her substance use, or child protection issues, or domestic violence, but that there was no immediacy of response within the system. So even for one service, they'd be saying, "That's really important. Can you come back at 10:00 o'clock tomorrow morning?" She wasn't going to come back at 10:00 o'clock tomorrow morning. You know? If there was a disclosure and a response, that needed to happen fairly quickly. I think again, we know that other referral pathways can take, you know, days, weeks or months. So, I think that immediacy... And that applies to,...not just to women, obviously, but that immediacy within the health response I think is key when you're dealing with issues with,...I suppose individuals that have a large degree of instability in their lives.” (Researcher2)

> “So, I think women in general were like, "Ohh, nobody ever does anything". There was a sense on the ground. It's like, "I've raised these issues so much and nobody actually ever *does* anything. Or they make phone calls, but nothing ever happens on the back of those. In relation to psychiatric services, there was, I think, a sense that people didn't listen, you know. Or there was an issue around say, like, "I don't get on with my psychiatrist, how am I meant to talk about these really personal things", or like, "the service provider has changed and now I have to go through the whole thing all over again.” (Researcher3)

Furthermore, to have available services and clear pathways in place does not imply that patients – especially those in great deals of emotional and/or physical pain – will have the trust or capacity to use them.

> So, I mean, and partly that's because I couldn't,...You know another researcher would go in and be like, "I managed to track this". And that's a good example because then you you're like, "OK, so we have this pathway and you come into your appointment and you..." But a woman's just after losing her baby, your drug use spirals. She's not gonna make that appointment, or she *might* not make that appointment and how do you build a flexible service that's around *care* in the acknowledgement that sometimes people are in so much pain that they find it difficult to go through the door. What does that level of flexibility look like? (Researcher3)

There was a general sense that resolving flaws in the system (e.g., availability of services, compartmentalised care, unresolved pathways) and reducing stigma through trauma-informed training will play a role in rebuilding trust and engagement. However, CEs noted a simultaneous need to increase empowerment by providing PEH a participatory space through which to take part in decision-making about health services. Traditionally, people in homelessness and addiction have little

freedom of choice. Often as a result, patients do not feel in charge of their own health or a sense of entitlement to anything better (Hospital1).

> “So, I'm thinking of my HIV outpatients or Hep C outpatients, because somebody makes them do it or prompts them to come and they kind of just do it to keep you happy, rather than them and being able to look after their health. And I think that's also - like, they can't usually choose what food they eat because it's all, you know, whatever. They can't choose their living environment. There's very little control over their health anyway. Like, I would never have somebody come in and go, "Oh, it's time for my cervical smear. I'm due my smear can I have it now?"
>
> *Interviewer: Sure I wouldn't do that either.*
>
> I mean, I wouldn't do that either. But or, you know, "Can I have my annual flu jab?" You know, all those things that I suppose you and I might do to look after our health.
>
> I always think that teeth is a really good example. So, if you look at people who are homeless, their teeth are usually in really bad condition and often they need dentures in their 20s, 30s. And that actually has loads of consequences for their health. But it's, that self-care where you're like, "OK, right, I have to brush my teeth and my parents always made me brush my teeth and I want to have my teeth still healthy when I'm 80 so I brush my teeth every day." You know, but their lives are so different that they don't brush their teeth. I think that's a good illustration of how much they're caught in moment-to-moment survival and how little emotional and all kinds of, you know, practical ability they have to invest in their future health.” (Hospital1)
>
> “Well listen, it's about real listening. Right? And you see for a lot of people who are addicted, they're spoken 'at': ‘What you need to do is go into this programme and this is the programme.’” (Psychotherapist1)
>
> “There’s maybe a sense of not being entitled to anything better. Often, a lot of the people we work with say, "Oh, I've brought this on myself and I'm so ashamed and I did this to myself." Which is really sad as well.” (Hospital2)
>
> “Again, the feedback: like the patients mostly love that there's somebody there just to have their back, you know. You're often an advocate for,...because busy teams like surgeons, they'll come around if someone's not doing what they tell them. They're like, "Well, I don't care. I have lots of other patients", and they'll just leave them. You know, so you try and be the advocate for the person and try and explain that, "Look, he doesn't really mean to behave like this, his only way of knowing how to deal with the situation was always to fight their corner". You know, a lot of people in homelessness, they've had to fight for everything in their lives since they were kids, be it food, clothes. So then when they come in to a hospital and they're being told what to do, their only way of dealing with it is fighting back. So you're just there to advocate as well. To just be the person to give them a voice.” (Hospital3)

Providing service users with opportunities to have ownership and self-expression over their care can be a mechanism for re-instilling a sense of responsibility and engagement.

> “I think any form of autonomy or self-expression that can be afforded to people can help. Even if you can't be given your own locked door property, I think giving a certain level of control over certain things,... I'm trying to think of an example of what I mean. The rules in the property, like different hostels feel slightly different, and the ones that have a better feel are the ones where it feels a little more collaborative between the people who are there to run it as a job and the people who are there to live there as their home. Whatever can be given as either a responsibility or a decision that needs to be made about the environment, whatever can be given to the people living there, even if it seems like smaller things. Some places have stuff painted on the walls or, one of the hostels has a big blackboard where you can write anything you want to write, and it's an expression of you as an individual on the wall, if you want there to be. There's a little bit of ownership. I know it's not an apartment, but that little bit of ownership with that little bit of self-expression, I think gives less of a feeling that you're just a number in a bed that nobody cares about and, in giving you a little bit of ownership of the property, there's also a little bit more responsibility taken by people to take care of the place, which obviously makes it a much more pleasant experience. It's very much either a spiral that goes up or a spiral that goes down. In the places where there is not that and then also a little bit of conflict kicks off, all of a sudden it can really go the other way. You know, suddenly there's a real "us and them" sort of dynamic, like "they hate us, and they don't like us," and I don't think, there isn't necessarily a huge difference in reality between one property and the other in terms of what's actually put into it in terms of resources, but the experience can be drastically different.
>
> Yeah, so, a little bit of self-expression and the self-esteem in having responsibility, if everything is taken away from you in terms of you being able to express yourself, you being able to take responsibility for your environment, then you can see why destructive behaviors come out in response. Even smaller things where there's a bit of, whatever can be handed back - not handed back - whatever can be maintained by the residents should be allowed to be. I think the same can be said of healthcare and decisions made in healthcare” (Homeless Health Services1)

Incorporating user voices will also improve the quality and effectiveness of services.

> “I do think including the people that the work is about will be a really big, big piece of it. So, hearing voices of people in homelessness and some of the asylum seekers and that would be really good. To know how they how they would see it working and have them kind of central to the whole idea. Interventions work much better if people actually *want* them.” (Hospital2)

CEs mentioned how well-versed people experiencing homelessness are in the system (Researcher3, Hospital3) and skills such as resourcefulness and creativity (Hospital1).

> “They're *so* resilient. I would be so long dead if I had to do a 10th of what they do so. They tend to be really community oriented. There's actually a huge sense of looking out for each other and minding each other. .. there's also a lot of creative problem solving. Like, literally, how would I survive with no money, nowhere to stay and nothing to eat? They can be really good at figuring out how to how to manage something. “ (Hospital1)

Despite this, there is a lack of data looking at how PEH would like their own healthcare in Ireland (Hospital2).

> "I would think that there's a gap in,...like we really don't have any data that looks at how people who are in homelessness, or in socially marginalised groups would like their healthcare? Or would think is the best way for their healthcare to be delivered. So that obviously, we're not just making all the decisions for them. That it has to be in the community, you know, perhaps some of them feel that actually hospital-delivered care is better for them. And then how that would work...what the barriers are to that working and how those could be met? Whether it's peer support or key working, whatever." (Hospital2)

Considerations moving forward include creating spaces for service users to provide feedback anonymously and without repercussion (Hospital1), creating opportunities for exchange between policymakers and service users (Addiction Services2, Social Care1), and accounting for potential literacy barriers (Hospital1) and the well-cited dilemma that, *"the individuals that need support the most, unfortunately, are the ones that may be least likely to engage." (*Social Care1)

> "I think there is a theoretical complaints line, but I think certainly before, it's a bit less now post Covid, but before you'd be allocated a different bed every night. So, you'd ring this thing called the free phone and you could ring at like 3 different times a day and all the beds were always gone and then the person who answered the phone would place, look up and see what's free, and place you. And they would know you and if you had complained about them, you mightn't get them to give you a good hostel. So, that would have been the fear, certainly. And a lot of our patients wouldn't be that literate, like literacy would be a major barrier to complaining about things." (Hospital1)

> "A lot of the management coming into the drug services today are managers of a business and they run it like a business. It's not people coming from the bottom up that would understand,... Very rare would you find someone who's come from the bottom of an organization up who would've worked the street level, who understood the whole homeless, hostels, polydrug use, needle exchanges, all that, and then moved into,... it's very rare. Very rare would they even be talking to people in homelessness. So basically, you've got business-type managers running the funding, going after the funding piece, and stats. You're not you anymore, you're P264 - single - white - female from Dublin South. We're looking for funding for you to go somewhere. So, you've stopped being a person who can contribute." (Addiction Services1)

> "Our management have their own connections but whether they influence policymakers and government bodies and stuff, I don't know. Because it's a much higher level. It's at the national level. I think people realise this, the government realises this, and then there's definitely more money poured into services such as ours or others, you know, residential or otherwise. But it's just not enough. I think it would be good for people at the top level to sit down with our clients, with people who are actually using the services." (Addiction Services2)

> "We always get feedback. We run a client, kind of forum piece. Where all of our clients will be offered to come on board. We've a chap, *[call him Jim]*, who'll sit with clients, he'll talk

about, "What did you find when you got here? How were you greeted?" So that's constantly in play. So, every client that we deal with, we offer them a laptop where they can go into a page and it's got like 30 questions on it which are relevant. "What's lacking in dual diagnosis? How did you find...?" Blah blah blah blah, housing piece. They fill it in and that's sent off and Joe does a recording. That's then sounded back into the system about where we're lacking, so it's client fed as it should be as opposed to 'from the top down'." (Addiction Services1)

"If the situation was complicated, it would be easy. It's *complex*. OK, so if you were to say to me, "*[Me]*, if you had a magic wand, what would you ask for?" I would say *engagement*. To get young people. Because the people,... they talk about hard-to-reach families and I know they talk about hard-to-reach services also. But what I *would* say is that the families that I come across that need support the most, unfortunately, are the ones that may be least likely to engage. You know, there's a whole load of different reasons for that, you know, depending on the family." (Social Care1)
