# Supplementary material for: Priority healthcare needs amongst people experiencing homelessness in Dublin, Ireland: A qualitative evaluation of community expert experiences and opinions
Source: PLoS One. 2023 Dec 14;18(12):e0290599. doi: 10.1371/journal.pone.0290599 (PMC10720995; doi:10.1371/journal.pone.0290599)
Supplement: S2 Text — (PDF) [file pone.0290599.s003.pdf]

UCD Connect

Carolyn Ingram <carolyn.ingram@ucd.ie>

LS-E-125-Ingram-Perrotta Low Risk Study

exemptions.ethics@ucd.ie <exemptions.ethics@ucd.ie> 27 June 2022 at 16:28
To: Carolyn Ingram <carolyn.ingram@ucd.ie>
Cc: Carla.perrotta@ucd.ie

Dear Carolyn

Thank you for your low risk study submission to the Human Research Ethics Committee – Sciences (HREC-LS) which meets the criteria for a low risk study. Should the nature of your research change and thereby alter your low risk status you will need to submit an application form for full ethics review. Please note for future correspondence regarding this low risk study that your Research Ethics Reference Number is: **LS-E-22-125-Ingram-Perrotta. This Low Risk Study is being approved on the condition that you observe the following:**

- **External REC Approval and/or Permission to Access/Recruit Human Participants/or their Data:** *(if applicable)* Please be aware that recruitment of participants or data collection should not begin until written permissions to access them are secured from external organisations/individuals/internal schools, colleges and units.

- **Researcher Duty of Care to Participants:** please ensure that ethical best practice is considered and applied to your research projects. You should ensure that participants are aware of what is happening to them and to their data whether a study is de-identified or not. All researchers have a duty of care to their participants who have the right to be informed, the right to consent to participate and the right to withdraw from the study.
- **Please note** that HREC no longer process **insurance cover** on behalf of the researcher. Researchers are required to complete a self-assessment form from the UCD SIRC office – please see www.ucd.ie/sirc/insurance/humanresearchinsurance

Any additional documentation should be emailed to research.ethics@ucd.ie quoting your assigned reference number (provided above) in the subject line of your email.

All Low Risk Studies are subject to a Research Ethics Compliance Review.

Regards

Jan

Janette Stokes

Research Ethics Officer

W. www.ucd.ie/research.ethics

From: Carolyn Ingram <carolyn.ingram@ucd.ie>
Sent: Monday 27 June 2022 14:40
To: Ethics Exemptions <exemptions.ethics@ucd.ie>
Subject: Application for ethical exemption - Anonymous Interviews

Dear colleagues at the Office of Research Ethics,

Please find attached an application for exemption from full ethical review for the first phase of my PhD research project, supervised by Dr. Carla Perrotta.

Please don't hesitate to contact us should you seek further clarifications.

Thank you for your time.

Kind regards,

Carolyn Ingram

--

Carolyn Ingram MPH

Research Assistant

School of Public Health, Physiotherapy and Sports Science
