# Supplementary material for: Priority healthcare needs amongst people experiencing homelessness in Dublin, Ireland: A qualitative evaluation of community expert experiences and opinions
Source: PLoS One. 2023 Dec 14;18(12):e0290599. doi: 10.1371/journal.pone.0290599 (PMC10720995; doi:10.1371/journal.pone.0290599)
Supplement: Attachment — Submitted filename: Response to Reviewer Comments.docx [file pone.0290599.s004.docx]

| **Editor Comments** | **Our Response** |
| --- | --- |
| 1. Please ensure that your manuscript meets PLOS ONE's style requirements, including those for file naming. | We have revised the manuscript’s headings, figure and table titles, and file naming in accordance with PLOS ONE’s style requirements. |
| 2. Please ensure that you include a title page within your main document. We do appreciate that you have a title page document uploaded as a separate file, however, as per our author guidelines (<http://journals.plos.org/plosone/s/submission-guidelines#loc-title-page>) we do require this to be part of the manuscript file itself and not uploaded separately. Could you therefore please include the title page into the beginning of your manuscript file itself, listing all authors and affiliations. | We have included a title page within the main document in accordance with PLOS ONE style requirements. |
| 3. We note that you have stated that you will provide repository information for your data at acceptance. Should your manuscript be accepted for publication, we will hold it until you provide the relevant accession numbers or DOIs necessary to access your data. If you wish to make changes to your Data Availability statement, please describe these changes in your cover letter and we will update your Data Availability statement to reflect the information you provide | We have revised our Data Availability statement and explained these changes in our cover letter. The statement now reads:  All relevant data are within the paper and its [Supporting Information](https://journals.plos.org/plosone/article?id=10.1371/journal.pone.0283979#sec019) files. Relevant data includes transcript excerpts justifying the results and quotations reported in our manuscript. Full interview transcripts cannot be made publicly available due to the sensitive nature of the data (small study sample from a small community expressing potentially sensitive viewpoints and details which could lead to participants’ identification by colleagues or clients).  We have prepared a file (Supplemental File 2) with data to support all study results that is publicly available with the manuscript. The file contains extensive transcript excerpts (60 pages, 33,000 words) that fully support our results/conclusions. We believe this is in line with PLOS ONE’s Data Availability policy for qualitative data, which states: “For studies analyzing data collected as part of qualitative research, authors should make excerpts of the transcripts relevant to the study available in an appropriate data repository, within the paper, or upon request if they cannot be shared publicly.” |
| 4. Please ensure that you include a title page within your main document. You should list all authors and all affiliations as per our author instructions and clearly indicate the corresponding author. | We have included a title page within the main document listing all authors and affiliations and indicated the corresponding author as per your author instructions. |
| 5. We note that [Figure 1] in your submission contain [map/satellite] images which may be copyrighted. All PLOS content is published under the Creative Commons Attribution License (CC BY 4.0), which means that the manuscript, images, and Supporting Information files will be freely available online, and any third party is permitted to access, download, copy, distribute, and use these materials in any way, even commercially, with proper attribution. For these reasons, we cannot publish previously copyrighted maps or satellite images created using proprietary data, such as Google software (Google Maps, Street View, and Earth). For more information, see our copyright guidelines: <http://journals.plos.org/plosone/s/licenses-and-copyright>.   We require you to either (1) present written permission from the copyright holder to publish these figures specifically under the CC BY 4.0 license, or (2) remove the figures from your submission: | In accordance with PLOS ONE’s Licenses and Copyright policy (<https://journals.plos.org/plosone/s/licenses-and-copyright>), we have provided a direct link to the source of the basemap we used to create the map in R and provide attribution to this source in the corresponding figure legend. We also provide information regarding the terms of use or license information for the map.  The new Fig 1 legend reads:  Map constructed by the authors using R version 4.1.3 leaflet package (R Foundation for Statistical Computing, Vienna, Austria). Map tiles by [Stamen Design](http://stamen.com/), under [CC BY 4.0](http://creativecommons.org/licenses/by/4.0). Map data by [OpenStreetMap](http://openstreetmap.org/), *open data* licensed under the [Open Data Commons Open Database License](https://opendatacommons.org/licenses/odbl/) (ODbL) by the [OpenStreetMap Foundation](https://osmfoundation.org/) (OSMF). |
| Reviewer 1 Comments | Our Response |
| For me, the paper is unusually structured, and in this regard there is room for revision... The reporting of findings begins in a conventional way on page 6 (using the authors' running footer manuscript page numbers), with two thematic headings ('State of homelessness...' and 'Healthcare usage...'). The next thematic heading, 'Priority healthcare needs', appears with an underlining which for me implies it has a thematically different status compared with the first two themes. This is not entirely clear, though. The 'Priority...' themes and sub-themes, with sample data extracts, are then displayed in a table, with recommendations alongside. This strikes me as an unusual way of presenting what might more usually be a rich, thematic, qualitative analysis. I also think that, at this point in the paper, it is far too early to be adding recommendations. I am also then somewhat thrown by the textual summary of these same tabulated themes, without data extracts, from page 15 onwards.  Overall, then, I think a more conventional presentation of thematic findings followed by a considered set of recommendations arising from these would work better and would also make for a more succinct read without the need to cross-refer the content of Table 3 and the textual summary beneath. An option here would be, I think, to avoid the use of Table 3 altogether. | Thank you, Reviewer 1, as this is a very helpful observation.  We have revised the format of Thematic headings so that the first three thematic headings ‘State of homelessness & addiction in Dublin, Healthcare usage & outcomes, and Priority healthcare needs’ have the same thematic status.  As suggested, we have removed Table 3 from the manuscript and added it as a Supplemental File. Before doing so, we cross-referenced the content of the tabulated themes with that of Table 3 to ensure that no information would be lost without presentation of the quotes. We added a quote from one community expert to Sub-theme 4.1 but otherwise found that no critical information was lost by removing the table.  A summary of considered recommendations is included in the Conclusion section. |
| As an aside, there needs to be some relabelling of figures, as Fig 2 is used in the text to refer both to the map of services and to the summary of themes. | We have made sure that each ‘Fig’ is referring to the correct object and revised Fig2 title for clarity. |
| **Reviewer 2 Comments** | **Our Response** |
| 1. Background: Since the article is centered on the concept of equity, I would appreciate a better understanding of equity issues in the drivers of the unhoused or in the provision of services that need to be addressed. For example, the authors state that substance use is the main cause of homelessness, but do not mention the structural and societal factors that contribute to substance use. Better defining what equity means in this context would be helpful. | Thank you Reviewer 2, this is a helpful suggestion. We have expanded our introduction paragraph to better define what equity means in the context of our research:  Rising homelessness levels across Europe have drawn increased attention to the health consequences of precarious housing. A health equity perspective – the belief that everyone has the right to a fair and just opportunity to attain their full potential for health – emphasizes that health disparities amongst people experiencing homelessness (PEH) are unnecessary and avoidable. Rather than the result of biological differences, they result from social and economic processes that create and recreate differences in access to housing and in turn health^1^. Low income and minority individuals and families are more vulnerable to the structural, institutional, relationship, and personal causes that drive homelessness^2^. Relationship breakdown as a result of domestic violence or abuse, or unemployment and financial stress, may leave someone without stable accommodation, as can the death of a family member^2^. Youth leaving foster care or those leaving prison or mental health facilities may have nowhere to go upon release or discharge^2^. People entering homelessness are more likely to have health issues, mental health issues, learning difficulties, problematic alcohol and drug use, or experiences of trauma in childhood^2^. In the Republic of Ireland, a country facing a severe housing crisis, structural causes exacerbate these housing inequalities by providing limited social housing and relying on the private rental market which drives rent spikes and insecure tenancies^2^.  Once in homelessness, an individuals’ health worsens and their life expectancy shortens^3^. PEH are at higher risk of premature mortality, disability, and chronic conditions and rely heavily on acute care ^4,5^. PEH in Ireland are at increased risk of substance misuse, self-harm, frequent emergency department (ED) presentation, and psychiatric admission. They are more likely to be hospitalised for venous thromboembolism (VTE), pneumonia/bronchitis, cellulitis, and seizure but less likely to complete their hospital admission ^5^. Despite facing adverse health outcomes, PEH experience reduced access to care due to a variety of external barriers including physical proximity to services; administrative barriers such as queues, appointment systems, and complicated application processes for care; and stigma and judgement from healthcare providers^6^. Once able to access services, PEH still may not use them due to competing priorities (e.g., finding food or a place to sleep), presumption of poor treatment, or negative emotions like fear, embarrassment, hopelessness, and poor self-esteem^6^.  Compounding housing and health inequalities stress the extent to which the individuals most likely to enter homelessness are the least equipped to combat its health consequences. In this context, achieving health equity will require not only addressing the downstream drivers of homelessness, but also improving equitable access to healthcare for PEH by strengthening health planning and the allocation of resources. This, first, requires understanding priority health concerns amongst PEH within a shared system^7^. |
| 2. Methods: While I agree that qualitative approaches are essential for understanding the needs of marginalised persons, I would recommend reframing the use of qualitative methods as something novel in needs assessments. Rather, most needs assessments are considered inadequate without the incorporation of community voices and perspectives on needs through qualitative engagement. There is considerable literature on how to do needs assessments that makes this clear. It would be interesting, for example, to include a review of the literature on how perspectives of the unhoused are incorporated into needs assessments, including reaching out to service providers. | Absolutely, yes, this is a great recommendation. We have revised our introduction to read:  Qualitative methods can provide timely insights into pressing health and social care needs as they can be conducted within a short time period and with involvement of community experts and members from marginalised groups^7,10^. Integrating qualitative techniques into local health needs assessments enables policymakers and healthcare providers to work out which interventions are needed most in their area; to plan strategically what services are needed in the future; to work out where resources should be focused based on evidence; and to address health inequalities ^11^. Guidelines specific to homeless health needs assessments recommend asking both people who are homeless as well as professionals who deliver services to people who are homeless to identify gaps in healthcare provision, barriers, and solutions^11^. Research teams from Calgary and San Francisco have demonstrated the efficiency and effectiveness of this approach, employing semi-structured interviews with staff in homeless health and service organisations to help identify priority health and healthcare needs amongst PEH across each city ^12,13^. |
| 3. Data Collection: The authors refer to the ethnographic study of the unhoused as part of the larger study and how this influenced the development of questions. This is very interesting, but if it is going to be included more information on how these two activities informed each other is important. Did the interview questions evolve over time as researchers gained more perspective on housing issues? Additionally, there is some confusion about the identification of interviewees since the data collection section describes being informed by the observations, while the recruitment refers to using a directory of service providers. | We have resolved these discrepancies as follows:  *RE: did the interview questions evolve over time as researchers gained more perspective on housing interviews?*  Revision: We based interview questions on World Health Organization Community Health Needs Assessment guidelines ^24^ and refined questions over time based on perspectives gained from other interviews and the lead researcher’s field work with homeless service users (Table 1). For example, after hearing PEH mention experiencing traumatic events after which they had nowhere to go/no one to turn to, we added, *“Have you spotted any gaps in services?”* Or, after multiple professionals in addiction services mentioned an overreliance on methadone, we added a question about methadone prescribing in subsequent interviews with primary care providers.  *RE: there is some confusion about the identification of interviewees…*  Revision: We identified potential CEs through (1) an internet search of homeless health and addiction services in Dublin, and (2) based on recommendations made by community partners working in the primary care and addiction service clinic where the lead researcher conducted her fieldwork. |
| 4. Results: The results reflect a comprehensive analysis and thematic coding framework. However, Table 3 needs to be revised. The length is a bit overwhelming. More importantly, the quotations included for some of the subthemes don’t really describe the name of the subthemes. The first sub theme for example, includes a quote that describes the need for a change in mindset but doesn’t mention health equity, thus it appears that the coders are making an interpretive leap. I would say the same for the theme strategic planning. It seems that the code is named more for the solution rather than the description of the issue. Since this is the results table, it is important to learn the perspectives of the service providers and where the commonality across different types of providers, perhaps. | Based on Reviewer 1’s suggestion and Reviewer 2’s feeling that Table 3 is too long, we have chosen to include it as a supplemental file. We have cross-checked table contents with the written results to ensure that this has not resulted in a loss of clarity or content. We do not believe it has. Within the – now supplemental – Table 3, we have chosen new quotes that better represent the names of themes/subthemes: **Promote Culture that Values Health Equity** \| *“The priority is not for our guys. Look at the housing crisis, or the homelessness crisis. With time, it’s not getting better, it’s getting worse. The policymakers, the parties in charge, society… we tend to prioritise the most wealthy. We have to start prioritising those most in need.” (Addiction Services 2)* Strategic Planning \| *“15 years I'm doing this now and there's been billions pumped into homeless and addiction services. I think there's about 55,000 people now working in the industry and yet, it's getting worse. It’s always reactive policies. It’s consistency we need and actually talking to people and thinking through long-term strategies.” (Addiction Services1)* Also, we now include as a supplemental file the transcript excerpts that justify all study results. Excerpts are colour coded by service provider category (i.e., health services, addiction services, etc) which allows for the comparison of responses across different types of providers. Should readers have any uncertainties regarding the data underlying specific findings, we hope this addition will be helpful. |
| In a related issue, the recommendation column and references to it belong in the discussion section. The recommendations are a response to the data and reflect the authors’ interpretation of practice and policy approaches to improve health equity for the unhoused. I would include a separate table, or perhaps just discuss recommendations in the discussion section. Since the paper is addressing very complex issues, this would also demonstrate how solutions work together to solve problems, rather than addressing one issue at a time. | Thank you. We have removed Table 3 and the associated recommendations column from the main manuscript. Upon re-examination of the manuscript, we find that that the Conclusion paragraph provides a sufficient summary of considered recommendations stemming from study findings. For the sake of conciseness, we have chosen not to add an additional Recommendations paragraph. We hope that the Conclusions drawn will leave readers with a sense of how solutions might work together to solve these complex problems. |
| 5. The discussion section is very informative and relates well to the literature on the subject. I would recommend a review of the article to ensure active voice in the methods and analysis description. The authors do a nice job describing each person’s role, but I always appreciate understanding who the authors are in relationship to the population and the study. | We have changed the article methods and descriptions to be in active voice. We like this too! Thank you again for your helpful feedback. |
